# Temporary Fertility Decline after Large Rubella Outbreak, Japan

**DOI:** 10.3201/eid2606.181718

**Published:** 2020-06

**Authors:** Kenji Mizumoto, Gerardo Chowell

**Affiliations:** Kyoto University, Kyoto, Japan, and Hokkaido University, Hokkaido, Japan (K. Mizumoto);; Georgia State University, Atlanta, GA, USA (K. Mizumoto, G. Chowell);; Fogarty International Center, National Institutes of Health, Bethesda, Maryland, USA (G. Chowell)

**Keywords:** outbreak, time series, birth risk, rubella, rubella virus, fushin, German measles, fertility, viruses, congenital rubella syndrome, CRS, miscarriage, stillbirth, birth defects

## Abstract

Japan experienced 2 large rubella epidemics in 2004 and 2012–2014. Because of suboptimal immunization levels, the country has been experiencing a third major outbreak during 2018–2020. We conducted time series analyses to evaluate the effect of the 2012–2014 nationwide rubella epidemic on prefecture-level natality in Japan. We identified a statistically significant decline in fertility rates associated with rubella epidemic activity and increased Google searches for the term “rubella.” We noted that the timing of fertility declines in 2014 occurred 9–13 months after peak rubella incidence months in 2013 in 4 prefectures with the highest rubella incidence. Public health interventions should focus on enhancing vaccination campaigns against rubella, not only to protect pregnant women from infection but also to mitigate declines in population size and birth rates.

Japan has recently experienced 2 large rubella epidemics. A 2004 epidemic had 4,248 reported cases, and another outbreak during 2012–2014 had 12,614 reported rubella cases and 45 reported cases of severe birth defects in newborns (*1**–**4*). Because of suboptimal immunization levels, Japan is now experiencing a third major rubella outbreak that began in 2018 (*5**–**7*). This epidemic has resulted in 5,296 reported cases as of April 12, 2020, and has affected all 47 prefectures by the 13th week of 2020 (*8*).

Rubella infections during early pregnancy can lead to serious health consequences, including miscarriages, stillbirths, and severe birth defects in newborns, known as congenital rubella syndrome (CRS) (*9*). The continued rubella epidemic in Japan led the US Centers for Disease Control and Prevention to issue a travel alert on October 22, 2018, that urged increased precautions and recommended pregnant women not protected against rubella avoid traveling to Japan (*9*).

Past studies have suggested that the 1918–1920 influenza pandemic had a profound effect on those planning to conceive children, which led to declines in birth rates after adjusting for maternal death (*10*,*11*). We examined the effects of Japan’s large rubella outbreak during 2012–2014 on behavioral changes among women of childbearing age by quantifying the temporal changes in fertility rates and rubella cases in 4 prefectures of Japan that experienced the brunt of the 2013 rubella epidemic.

## Methods

### Data Sources

We extracted data on births/month and estimated female population/year stratified by age and prefecture from January 1, 2013–December 31, 2017, for Japan and 4 prefectures with the highest cumulative number of rubella cases in 2013 from the Ministry of Internal Affairs and Communications in Japan (*12*). Then, we calculated monthly estimates of the female population 15–49 years of age during 2013–2018 by using a smoothing cubic spline interpolation-fitting method with a knot at each data point. Because the number of births/age group was not available, we considered the fertility rate to be the proportion of births divided by the number of female persons 15–49 years of age/1,000 population/year.

We collected notifications of rubella and CRS cases from weekly reports published by the National Institute of Infectious Diseases in Japan and converted these to monthly case counts (*8*). Surveillance relies on mandatory notifications and medical institutions must report all diagnosed rubella and CRS cases. Clinical diagnosis of rubella includes symptoms of generalized rash, swollen lymph nodes, and fever.

We also extracted relevant monthly data on Google search terms from Google Trends (https://trends.google.com) to quantify the public attention to the rubella epidemic. We suspect anxiety was driven by a perceived risk rather than actual risk. For example, during a 2014–15 outbreak of Ebola virus, the public conducted internet searches to collect information related to the epidemic, even in areas with extremely low risk for infection (*13*). Google Trends uses searches for keywords and search terms to provide data on search volume in a geographic region over time and generates an output scale of 0–100 (*14*). 

We conducted a search of Google Trends by using the search topic strategy for January 1, 2012–December 31, 2017, and included the Tokyo, Kanagawa, Osaka, and Hyogo prefectures by using the Japanese word for rubella, “fushin.” We used data from years after 2013 to exclude demographic changes and perinatal outcomes associated with the Great East Japan Earthquake that occurred on March 11, 2011 (*15*).

### Statistical Analysis

Time series data can be decomposed into three components: seasonality, long-term trends, and random (*16*,*17*). For our study, the seasonal and trend components contain effects of long-term changes. Temporal variation in the fertility rate over time can be effected by systematic seasonal changes, such as the number of marriages, and long-term trends, such as population demography. By filtering out these components, we were able to concentrate on a single event. We applied moving averages to extract monthly seasonal- and trend-adjusted elevated fertility rates and their residuals. To detect temporal associations between these time series data, we examined cross-coefficients of fertility rates, rubella cases, and Google searches for “rubella” at different lag intervals (lags or leads <12 months) for each combination of 2/3 variables. A more detailed description of this procedure is published elsewhere (*10*,*18*,*19*). Then, we used an augmented Dickey-Fuller test for stationarity analysis. We used the Mann-Kendall test for trend analysis and null hypotheses of 0 cross-correlation for each of the estimated correlation coefficients.

We used a bootstrap method with 1,000 replications to calculate mean monthly and annual fertility rates in 2014 and corresponding confidence intervals. We performed statistical analyses in R version 3.2.3 (R Foundation for Statistical Computing, https://www.r-project.org).

## Results

**Figure 1:**
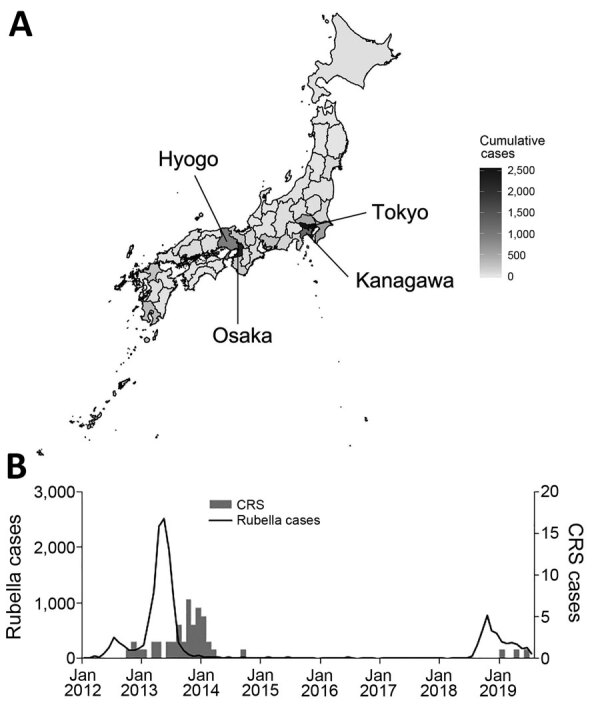
Spatial and temporal variations of rubella and CRS in Japan, 2013–2019. A) Geospatial variation in cumulative rubella cases by prefecture, 2013. B) Temporal distribution of rubella and CRS cases by month during January 1, 2012–July 31, 2019. Black line indicates number of cases of rubella. Gray bars indicate number of cases of CRS. CRS, congenital rubella syndrome.

We assessed geospatial variation in cumulative rubella cases across Japan in 2013 (Figure 1, panel A). The highest rubella incidence occurred in Tokyo (2,547 cases), Osaka (2,241 cases), Kanagawa (1,230 cases), and Hyogo (941 cases). The distribution of cumulative rubella cases across all prefectures was highly skewed to the right with a median of 44.0 (interquartile range 20.5–106.5). We assessed the number of rubella and CRS cases by month during 2013–2019. During the 2012–2014 outbreak, the highest incidence of rubella was 2,513 cases in May 2013 and the highest incidence of CRS was 7 cases in October 2013 (Figure 1, panel B).

**Figure 2:**
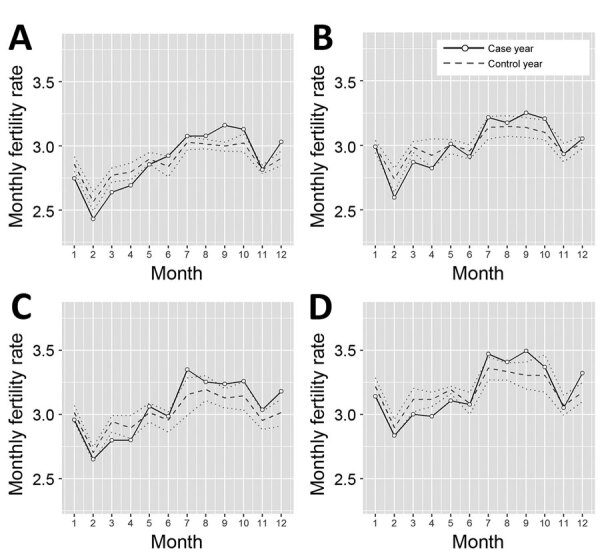
Temporal variation of fertility rates by prefecture in Japan, 2013–2017. A) Tokyo; B) Kanagawa; C) Osaka; and D) Hyogo. Solid line indicates average fertility rate during the case year, 2014. Dashed line indicates the average fertility rate during the combined control years, 2013, 2015, 2016, and 2017. Dotted lines indicate upper and lower limits of 95% CI for control years.

We assessed the seasonal variation of fertility rates for the 4 prefectures with the highest rubella burden (Figure 2). We observed fertility rate declines for 2014 during 5 months in Tokyo, 2 months in Kanagawa, 3 months in Osaka, and 4 months in Hyogo, after which rates increased during 6 months in Tokyo, 5 months in Kanagawa, 2 months in Osaka, and 4 months in Hyogo. Of note, fertility rate declines in 2014 occurred in the first half of the year, which corresponds to 9–13 months after rubella incidence peaked in Tokyo, 11–12 months after peak incidence in Kanagawa, 9–11 months after peak incidence in Osaka, and 8–12 months after peak incidence in Hyogo (Table). We believe behavioral changes occurred around the peak rubella incidences and calculated times after the peak incidence of rubella cases.

**Table:** Summary results on the timing of peak rubella cases and deficit fertility rates for 4 prefectures in Japan, 2013*

Prefecture			Peak month for Google searches, 2013	Decreased fertility, 2014			Lag time troughs, mo.	
		Month of lowest birth rates	Deficit rate at lowest month†	Peak rubella incidence–fertility rate decline	Peak Google search–fertility rate decline
Rubella case peaks, 2013					
Month	No. cases				
Tokyo	Apr	571	Apr	Mar	–0.090		11	11
Kanagawa	Apr	305	Apr	Mar	–0.130		11	11
Osaka	May	730	May	Feb	–0.120		9	9
Hyogo	May	260	May	Apr	–0.111		11	11

*Data on Google searches collected from Google Trends (https://trends.google.com). †Irregular only.

**Figure 3:**
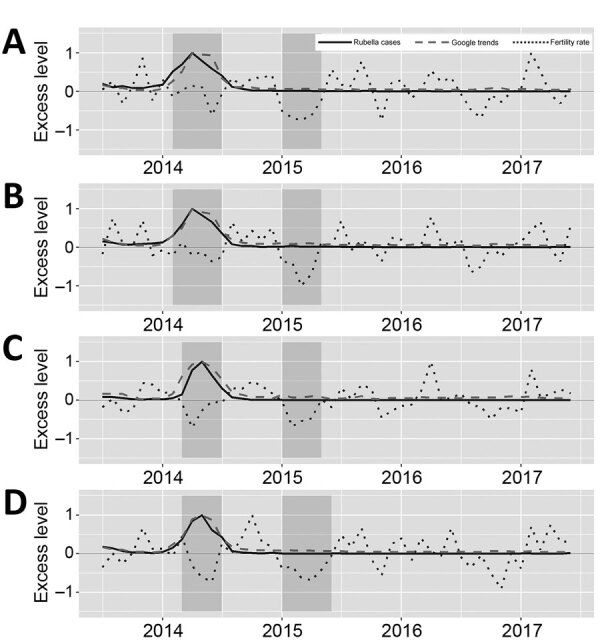
Cross-correlation between rubella cases, Google searches for “rubella,” and elevated fertility rates, Tokyo, Japan, 2012–2016. Cross-correlation coefficients were calculated in each lag, –12 months, lead period, +12 months, and at 0. Bars indicate cross-correlation coefficients between A) fertility rate and rubella case time-series; B) fertility rate and Google searches for “rubella” time-series; and C) rubella cases and Google searches for “rubella” time series. Horizontal dashed lines are the confidence limits (upper limit, 0.28; lower limit, –0.28) for the null hypothesis of 0 true cross-correlation coefficients between the 2 time-series. Google search data collected from Google Trends (https://trends.google.com).

**Figure 4:**
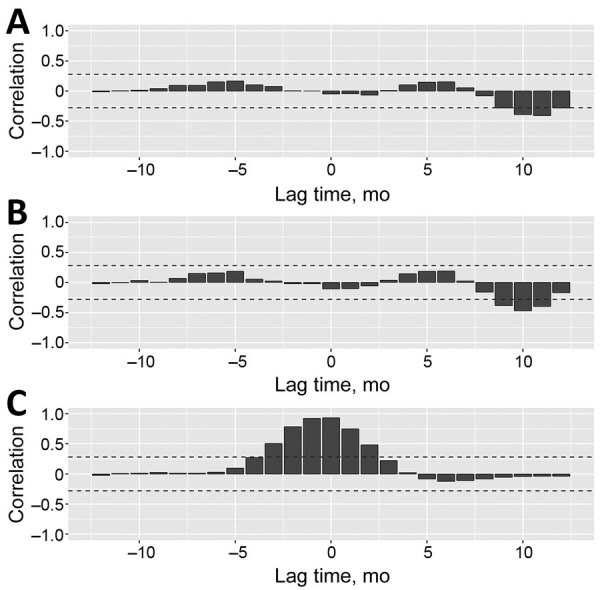
Temporal distribution of rubella cases, Google searches for the term “rubella,” and elevated fertility rates by prefecture, 2013–2017, Japan. A) Tokyo; B) Kanagawa; C) Osaka; D) Hyogo Time-series of rubella cases, Google searches for the term “rubella,” and seasonally- and trend-adjusted elevated fertility rates. The data are scaled between –1 and 1. Dark gray areas show the peak timing of rubella cases and the corresponding drop timing of fertility rates. Google search data collected from Google Trends (https://trends.google.com)..

We plotted the time series of rubella cases, Google searches for “rubella,” and seasonality- and trend-adjusted fertility rates for the 4 prefectures with the highest rubella incidence (Figure 3). We found that peaks in rubella cases and Google searches for “rubella” were synchronized across the 4 prefectures with the most rubella cases. However, troughs in elevated fertility rates occurred 9–11 months after the peak in rubella cases and Google searches for “rubella” (Table). To statistically assess the timescale of the association between rubella cases, Google searches for “rubella,” and elevated fertility rates, we calculated cross-correlations between these 3 signals in the time series. In Tokyo, we found a negative association between rubella incidence and fertility rates at a time lag of 9 months (r = –0.280), 10 months (r = –0.390), 11 months (r = –0.408), and 12 months (r = –0.284) and between Google searches for “rubella” and fertility rates at time lags of 9 months (r = –0.382), 10 months (r = –0.468), and 11 months (r = –0.397) (Figure 4; Appendix Table 1). We also identified negative associations between rubella incidence and fertility rates in Kanagawa at time lags of 9 months (r = –0.320), 10 months (r = –0.428), and 11 months (r = –0.446) and between Google searches for “rubella” and fertility rates at time lags of 9 months (r = –0.385), 10 months (r = –0.461), and 11 months (r = –0.432) (Appendix Table 2, Figure 1). In Osaka, we found positive and negative associations between rubella cases and fertility rates at a time lag of 5 months (r = 0.296), 9 months (r = –0.365), 10 months (r = –0.424), and 11 months (r = –0.319) and between Google searches for “rubella” and fertility rates at 4 months (r = 0.286), 5 months (r = 0.316), 9 months (r = –0.356), 10 months (r = –0.437), and 11 months (r = –0.361) (Appendix Table 3, Figure 2). In Hyogo, we identified positive and negative associations between rubella cases and fertility rates at a time lag of 5 months (r = 0.347), 9 months (r = –0.309), 10 months (r = –0.410), and 11 months (r = –0.402) and between Google searches for “rubella” and fertility rates at 0 months (r = –0.298), 5 months (r = 0.346), 9 months (r = –0.310), 10 months (r = –0.398), and 11 months (r = –0.393) (Appendix Table 4, Figure 3). We observed several positive associations between rubella incidence and Google searches for “rubella” in all 4 prefectures.

We decomposed time series data of fertility rates for Tokyo, Kanagawa, Osaka, and Hyogo (Appendix Figure 4). We observed downward trends in fertility rates but a small peak during 2015 and an upward trend before 2015 in Tokyo. Seasonally, fertility rates tend to decrease in the first half of the year and increase in the second half of the year.

We did not find statistically significant differences in fertility rates between 2014 and the control years. Fertility rate differences between 2014 and control years were 34.87 (95% CI 34.01–35.20)/1,000 women of childbearing age in Tokyo, 36.52 (95% CI 35.06–36.92)/1,000 women of childbearing age in Kanagawa, 35.95 (95% CI 35.25–36.6)/1,000 women of childbearing age in Osaka, and 37.97 (95% CI 37.15–38.53)/1,000 women of childbearing age in Hyogo.

## Discussion

We assessed the effect of the 2012–2014 rubella epidemic on prefecture-level natality in Japan. We identified a statistically significant decline in fertility rates associated with rubella epidemic activity and Google searches for “rubella.” We noted declines in fertility rates in 2014 that occurred after each peak month of rubella case incidence in 2013, which was 9–13 months after peaks in Tokyo, 11–12 months in Kanagawa, 9–11 months in Osaka, and 8–12 months in Hyogo. Considering the relatively small number of rubella cases during 2012–2014, we do not think the reduction in fertility was caused by miscarriages or stillbirths but by voluntary pregnancy delays because of perceived risk for CRS, which is reflected in increases in Google searches for “rubella.”

Birth rate declines associated with infectious disease epidemics could exacerbate the effects of demographic changes in Japan, which are characterized by decreased population, declines in birth rates, and a delayed childbearing trend. According to the 2010 census, the population of Japan is ≈127.09 million, a decrease of 962,607 from the previous census (*20*). In addition, the fertility rate was 1.44/1,000 female persons 15–49 years of age in 2016, much lower than the replacement-level fertility of 2.1/1,000, the minimum level needed to sustain population size (*21*). Similarly, the average age of women at first childbirth rose from 25.6 years of age in 1970 to 30.7 years of age in 2016. Older maternal age is associated with increased risks for adverse pregnancy and birth outcomes (*22**–**26*). 

We found no statistically significant differences in birth rates between 2014 and the control years (2013, 2015, 2016, and 2017). However, pregnancy delays related to fear of infection and the increased proportion of late-age childbearing exacerbate the declining birth trend for the ongoing and future rubella epidemics. Therefore, Japan’s government should implement public health interventions to rapidly curtail rubella transmission, protect childbearing mothers, and sustain birth rates.

Our study has several limitations. First, we did not establish a causal relationship between an increase of rubella cases and a decrease of fertility rate, but studies have suggested a person’s behavior will change not only because of actual risk but also because of perceived risk (*13*). Indeed, some segments of the population with low vaccination coverage in Japan, such as women 24–34 years of age who are part of a relatively small group of persons born during 1989–1993 in which only 78.3% are seropositive, presumably worry about infection and CRS (*1*,*6*). In addition, soon after the rubella outbreak occurred, the Ministry of Health, Labour and Welfare issued its first notice to alert the public on May 25, 2012, and subsequently issued an additional 7 notices during the outbreak (*27*). On January 29, 2013, Japan’s National Institute of Infectious Diseases published specific guidelines for prevention and control of rubella and CRS (*28*). These approaches likely increased the population’s awareness about the risk for rubella infection and its potential consequences. Furthermore, a substantial number of pregnancies likely were delayed until the end of the outbreaks because of concerns about rubella infection, and public awareness, which is partially supported by the internet search activities recorded by Google Trends. In addition, the relationship between the 1918 influenza pandemic and fertility, in which a direct causal relationship was estimated (*10*,*18*,*19*), indicates a remarkable decline in births occurring 9–11 months after the surge in pandemic mortality rates. A similar phenomenon was observed for the Zika virus (ZIKV) epidemic. After the identification of the probable association between ZIKV infection during pregnancy and microcephaly in 2016 (*29**–**31*), public concern over ZIKV quickly increased in Brazil, where a substantial number of microcephaly cases initially were reported. As a result, birth rates in Brazil’s largest cities during the second half of 2016 exhibited substantial reductions ≈9 months after the start of media coverage for the ZIKV epidemic (*32*,*33*). We found a similar fertility decline 9–11 months after the peaks in rubella case incidence and peak Google searches for “rubella” across all 4 prefectures. To reinforce the statistical association, we conducted additional analyses. We used influenza, which does not affect people’s fertility decisions, as a control and did not find a decline in fertility associated with influenza case counts or the Google searches for “influenza” (Appendix Tables 5–8, Figures 5–8). We explored wavelet cross-correlation to measure similarity between 2 signals at different scales (*34**,**35*). We identified relatively high coefficients at a lag time of 9–11 months in levels 2 and 3 between rubella incidence and fertility, and between rubella incidence and Google searches for “rubella,” consistent with our results (Appendix Tables 9–11, Figures 9–20).

Second, we did not identify the causal relationship for the substantial fertility rate increases ahead of substantial fertility rate declines. Although we examined the association between fertility rates and other factors, including economic index, such as prefectural-level unemployment rates and the Nikkei Index, and the number of marriages (data not shown), we did not identify statistically significant associations. Several interrelated factors likely played a role. In addition, we only observed the lagged increases in Osaka and Hyogo, and the number of the months and the correlation coefficients are relatively small compared with the lagged decrease.

Third, an increase in Google searches for “rubella” might have preceded an outbreak of rubella cases at the prefectural level because media reporting about the outbreaks raised public awareness, potentially leading to large-scale behavior changes in the country. However, the temporal variations of rubella cases and Google searches for “rubella” in all 4 prefectures are highly correlated at a time lag of 0 (Figure 4; Appendix Tables 1–4, Figures 1–3) and rubella case incidence and Google searches for “rubella” mostly were synchronized.

In conclusion, our analyses indicate a substantial temporal decline in the fertility rate in Japan after a major rubella outbreak in 2012–2014. Public health interventions should focus on enhancing vaccination campaigns against rubella, not only to protect pregnant women from infection but also to mitigate declines in population size and birth rates.

Appendix Additional information on temporary fertility decline after a large rubella outbreak, Japan.
