# Supplementary material for: Temporary Fertility Decline after Large Rubella Outbreak, Japan
Source: Emerg Infect Dis. 2020 Jun;26(6):1122–9. doi: 10.3201/eid2606.181718 (PMC7258466; doi:10.3201/eid2606.181718)
Supplement: Appendix — Additional information on temporary fertility decline after a large rubella outbreak, Japan. [file 18-1718-Techapp-s1.pdf]

Article DOI: https://doi.org/10.3201/eid2606.181718

Temporary Fertility Decline after a Large Rubella Outbreak, Japan

Appendix

Appendix Table 1. Cross-correlation coefficients between rubella cases, Google Trends, and elevated fertility rates, 2012–2018, Tokyo, Japan*

Lag time, mo	Rubella cases vs. fertility rates		Google Trends vs. fertility rates		Rubella cases vs. Google Trends	
	Correlation coefficient	Std. error	Correlation coefficient	Std. error	Correlation coefficient	Std. error
–12	–0.014	0.33	–0.022	0.33	–0.023	0.33
–11	0.004	0.33	–0.004	0.33	0.006	0.33
–10	0.013	0.32	0.031	0.32	0.013	0.32
–9	0.042	0.32	0.006	0.32	0.026	0.32
–8	0.095	0.32	0.070	0.32	0.011	0.32
–7	0.093	0.31	0.148	0.31	0.013	0.31
–6	0.153	0.31	0.161	0.31	0.029	0.31
–5	0.168	0.30	0.186	0.30	0.100	0.30
–4	0.105	0.30	0.055	0.30	0.271	0.30
–3	0.079	0.30	0.022	0.30	**0.506**	0.30
–2	0.006	0.29	–0.018	0.29	**0.787**	0.29
–1	–0.001	0.29	–0.019	0.29	**0.926**	0.29
0	–0.048	0.29	–0.105	0.29	**0.931**	0.29
1	–0.044	0.29	–0.102	0.29	**0.749**	0.29
2	–0.066	0.29	–0.059	0.29	**0.482**	0.29
3	0.009	0.30	0.037	0.30	0.224	0.30
4	0.102	0.30	0.144	0.30	0.019	0.30
5	0.150	0.30	0.186	0.30	–0.083	0.30
6	0.154	0.31	0.189	0.31	–0.122	0.31
7	0.053	0.31	0.023	0.31	–0.111	0.31
8	–0.081	0.32	–0.160	0.32	–0.084	0.32
9	**–0.280**	0.32	**–0.382**	0.32	–0.055	0.32
10	**–0.390**	0.32	**–0.468**	0.32	–0.040	0.32
11	**–0.408**	0.33	**–0.397**	0.33	–0.036	0.33
12	**–0.284**	0.33	–0.170	0.33	–0.037	0.33

*Bold text indicates statistical significance. Data on Google searches for the term “rubella” collected from Google Trends (https://trends.google.com).

Appendix Table 2. Cross-correlation coefficients between rubella cases, Google Trends, and elevated fertility rates, 2012–2018, Kanagawa, Japan*

	Rubella cases vs. fertility rates		Google Trends vs. fertility rates		Rubella cases vs. Google Trends	
Lag time, mo	Correlation coefficient	Std. error	Correlation coefficient	Std. error	Correlation coefficient	Std. error
–12	–0.015	0.33	–0.012	0.33	–0.002	0.33
–11	0.026	0.33	0.003	0.33	0.020	0.33
–10	0.082	0.32	0.112	0.32	0.026	0.32
–9	0.129	0.32	0.138	0.32	0.027	0.32
–8	0.221	0.32	0.222	0.32	0.011	0.32
–7	0.204	0.31	0.213	0.31	0.004	0.31
–6	0.162	0.31	0.148	0.31	0.021	0.31
–5	0.115	0.30	0.090	0.30	0.077	0.30
–4	0.006	0.30	–0.047	0.30	0.210	0.30
–3	–0.067	0.30	–0.086	0.30	**0.434**	0.30
–2	–0.100	0.29	–0.153	0.29	**0.717**	0.29
–1	–0.114	0.29	–0.132	0.29	**0.917**	0.29
0	–0.164	0.29	–0.187	0.29	**0.979**	0.29
1	–0.129	0.29	–0.119	0.29	**0.806**	0.29
2	–0.076	0.29	–0.032	0.29	**0.530**	0.29
3	0.051	0.30	0.080	0.30	0.236	0.30
4	0.189	0.30	0.232	0.30	0.018	0.30
5	0.214	0.30	0.199	0.30	–0.092	0.30
6	0.189	0.31	0.198	0.31	–0.127	0.31
7	0.078	0.31	0.029	0.31	–0.109	0.31
8	–0.082	0.32	–0.123	0.32	–0.063	0.32
9	**–0.320**	0.32	**–0.385**	0.32	–0.021	0.32
10	**–0.428**	0.32	**–0.461**	0.32	–0.006	0.32
11	**–0.446**	0.33	**–0.432**	0.33	–0.009	0.33
12	–0.272	0.33	–0.238	0.33	–0.022	0.33

*Bold text indicates statistical significance. Data on Google searches for the term “rubella” collected from Google Trends (https://trends.google.com).

Appendix Table 3. Cross-correlation coefficients between rubella cases, Google Trends, and elevated fertility rates, 2012–2018, Osaka, Japan*

	Rubella cases vs. fertility rates		Google Trends vs. fertility rates		Rubella cases vs. Google Trends	
Lag time, mo	Correlation coefficient	Std. error	Correlation coefficient	Std. error	Correlation coefficient	Std. error
–12	–0.020	0.33	–0.048	0.33	–0.028	0.33
–11	–0.046	0.33	–0.052	0.33	–0.014	0.33
–10	–0.083	0.32	–0.081	0.32	–0.014	0.32
–9	–0.108	0.32	–0.115	0.32	–0.008	0.32
–8	–0.090	0.32	–0.049	0.32	–0.029	0.32
–7	–0.016	0.31	0.034	0.31	–0.056	0.31
–6	0.167	0.31	0.136	0.31	–0.068	0.31
–5	0.266	0.30	0.238	0.30	–0.047	0.30
–4	0.178	0.30	0.207	0.30	0.030	0.30
–3	0.042	0.30	0.050	0.30	0.215	0.30
–2	–0.122	0.29	–0.130	0.29	**0.509**	0.29
–1	–0.278	0.29	–0.242	0.29	**0.800**	0.29
0	–0.263	0.29	–0.259	0.29	**0.952**	0.29
1	–0.099	0.29	–0.175	0.29	**0.870**	0.29
2	0.027	0.29	0.007	0.29	**0.573**	0.29
3	0.163	0.30	0.169	0.30	0.231	0.30
4	0.271	0.30	**0.286**	0.30	0.000	0.30
5	**0.296**	0.30	**0.316**	0.30	–0.113	0.30
6	0.240	0.31	0.263	0.31	–0.142	0.31
7	0.062	0.31	0.065	0.31	–0.109	0.31
8	–0.153	0.32	–0.156	0.32	–0.057	0.32
9	**–0.365**	0.32	**–0.356**	0.32	–0.027	0.32
10	**–0.424**	0.32	**–0.437**	0.32	–0.021	0.32
11	**–0.319**	0.33	**–0.361**	0.33	–0.025	0.33
12	–0.149	0.33	–0.202	0.33	–0.030	0.33

*Bold text indicates statistical significance. Data on Google searches for the term “rubella” collected from Google Trends (https://trends.google.com).

Appendix Table 4. Cross-correlation coefficients between rubella cases, Google Trends, and elevated fertility fates, 2012–2018, Hyogo, Japan*

	Rubella cases vs. fertility rates		Google Trends vs. fertility rates		Rubella cases vs. Google Trends	
Lag time, mo	Correlation coefficient	Std. error	Correlation coefficient	Std. error	Correlation coefficient	Std. error
–12	–0.041	0.33	–0.043	0.33	0.004	0.33
–11	–0.056	0.33	–0.077	0.33	0.038	0.33
–10	–0.103	0.32	–0.087	0.32	0.059	0.32
–9	–0.089	0.32	–0.094	0.32	0.053	0.32
–8	–0.016	0.32	–0.012	0.32	0.012	0.32
–7	0.064	0.31	0.103	0.31	–0.024	0.31
–6	0.175	0.31	0.181	0.31	–0.040	0.31
–5	0.188	0.30	0.198	0.30	–0.014	0.30
–4	0.143	0.30	0.129	0.30	0.083	0.30
–3	0.075	0.30	0.087	0.30	**0.285**	0.30
–2	–0.005	0.29	–0.033	0.29	**0.593**	0.29
–1	–0.156	0.29	–0.169	0.29	**0.879**	0.29
0	–0.257	0.29	**–0.298**	0.29	**0.981**	0.29
1	–0.268	0.29	–0.246	0.29	**0.843**	0.29
2	–0.103	0.29	–0.107	0.29	**0.538**	0.29
3	0.121	0.30	0.101	0.30	0.240	0.30
4	0.258	0.30	0.274	0.30	0.009	0.30
5	**0.347**	0.30	**0.346**	0.30	–0.092	0.30
6	0.265	0.31	0.269	0.31	–0.126	0.31
7	0.086	0.31	0.067	0.31	–0.115	0.31
8	–0.118	0.32	–0.116	0.32	–0.086	0.32
9	**–0.309**	0.32	**–0.310**	0.32	–0.060	0.32
10	**–0.410**	0.32	**–0.398**	0.32	–0.039	0.32
11	**–0.402**	0.33	**–0.393**	0.33	–0.038	0.33
12	–0.254	0.33	–0.263	0.33	–0.037	0.33

*Bold text indicates statistical significance. Data on Google searches for the term “rubella” collected from Google Trends (https://trends.google.com).

Appendix Table 5. Cross-correlation coefficients between influenza cases, Google Trends, and elevated fertility rates, 2012–2018, Tokyo, Japan*

	Influenza cases vs. fertility rates		Google Trends vs. fertility rates		Influenza cases vs. Google Trends	
Lag time, mo	Correlation coefficient	Std. error	Correlation coefficient	Std. error	Correlation coefficient	Std. error
–12	–0.093	0.33	–0.098	0.33	**0.354**	0.33
–11	–0.168	0.33	–0.227	0.33	**0.376**	0.33
–10	–0.053	0.32	–0.157	0.32	**0.329**	0.32
–9	–0.094	0.32	–0.187	0.32	0.079	0.32
–8	–0.099	0.32	–0.156	0.32	–0.144	0.32
–7	–0.039	0.31	–0.015	0.31	–0.271	0.31
–6	0.012	0.31	0.067	0.31	–0.335	0.31
–5	0.001	0.3	0.051	0.3	–0.354	0.3
–4	0.069	0.3	0.129	0.3	–0.384	0.3
–3	0.155	0.3	0.157	0.3	–0.308	0.3
–2	0.073	0.29	–0.004	0.29	–0.089	0.29
–1	–0.112	0.29	–0.059	0.29	**0.467**	0.29
0	0.043	0.29	0.065	0.29	**0.914**	0.29
1	–0.143	0.29	–0.117	0.29	**0.528**	0.29
2	–0.222	0.29	–0.183	0.29	0.19	0.29
3	–0.062	0.3	–0.043	0.3	–0.001	0.3
4	0.123	0.3	0.093	0.3	–0.18	0.3
5	0.077	0.3	0.103	0.3	–0.323	0.3
6	0.117	0.31	0.097	0.31	–0.391	0.31
7	0.117	0.31	0.082	0.31	–0.346	0.31
8	–0.062	0.32	–0.056	0.32	–0.277	0.32
9	–0.114	0.32	–0.097	0.32	–0.161	0.32
10	–0.004	0.32	–0.021	0.32	0.006	0.32
11	0.061	0.33	0.006	0.33	0.183	0.33
12	–0.083	0.33	–0.137	0.33	**0.377**	0.33

*Bold text indicates statistical significance. Data on Google searches for the term “influenza” collected from Google Trends (https://trends.google.com).

Appendix Table 6. Cross-correlation coefficients between influenza cases, Google Trends, and elevated fertility rates, 2012–2018, Kanagawa, Japan*

	Influenza cases vs. fertility rates		Google Trends vs. fertility rates		Influenza cases vs. Google Trend	
Lag time, mo	Correlation coefficient	Std. error	Correlation coefficient	Std. error	Correlation coefficient	Std. error
–12	–0.089	0.33	–0.076	0.33	**0.358**	0.33
–11	–0.156	0.33	–0.163	0.33	**0.362**	0.33
–10	–0.073	0.32	–0.155	0.32	0.304	0.32
–9	–0.196	0.32	–0.265	0.32	0.079	0.32
–8	–0.133	0.32	–0.144	0.32	–0.137	0.32
–7	0.005	0.31	–0.032	0.31	–0.258	0.31
–6	0.015	0.31	0.054	0.31	**–0.316**	0.31
–5	0.148	0.3	0.125	0.3	**–0.332**	0.3
–4	0.092	0.3	0.081	0.3	**–0.371**	0.3
–3	0.079	0.3	0.062	0.3	–0.299	0.3
–2	0.09	0.29	0.029	0.29	–0.099	0.29
–1	–0.039	0.29	0.006	0.29	**0.431**	0.29
0	–0.074	0.29	0.007	0.29	**0.916**	0.29
1	–0.153	0.29	–0.071	0.29	**0.507**	0.29
2	–0.204	0.29	–0.157	0.29	0.163	0.29
3	–0.078	0.3	–0.015	0.3	0.016	0.3
4	0.208	0.3	0.184	0.3	–0.139	0.3
5	0.109	0.3	0.098	0.3	–0.286	0.3
6	0.084	0.31	0.089	0.31	**–0.35**	0.31
7	0.037	0.31	0.007	0.31	**–0.313**	0.31
8	–0.101	0.32	–0.061	0.32	–0.255	0.32
9	–0.034	0.32	–0.043	0.32	–0.153	0.32
10	0.014	0.32	–0.031	0.32	–0.015	0.32
11	0.065	0.33	0.024	0.33	0.143	0.33
12	–0.055	0.33	–0.057	0.33	**0.334**	0.33

*Bold text indicates statistical significance. Data on Google searches for the term “influenza” collected from Google Trends (https://trends.google.com).

Appendix Table 7. Cross-correlation coefficients between influenza cases, Google Trends, and elevated fertility rates, 2012–2018, Osaka, Japan*

	Influenza cases vs. fertility rates		Google Trends vs. fertility rates		Influenza cases vs. Google Trends	
Lag time, mo	Correlation coefficient	Std. error	Correlation coefficient	Std. error	Correlation coefficient	Std. error
–12	–0.016	0.33	0.028	0.33	**0.329**	0.33
–11	–0.073	0.33	–0.1	0.33	**0.338**	0.33
–10	–0.035	0.32	–0.157	0.32	0.273	0.32
–9	–0.225	0.32	–0.299	0.32	0.06	0.32
–8	–0.079	0.32	–0.11	0.32	–0.131	0.32
–7	0.036	0.31	–0.016	0.31	–0.25	0.31
–6	0.049	0.31	0.091	0.31	–0.306	0.31
–5	0.096	0.3	0.108	0.3	–0.323	0.3
–4	0.01	0.3	0.09	0.3	–0.375	0.3
–3	0.128	0.3	0.184	0.3	–0.313	0.3
–2	0	0.29	0.016	0.29	–0.092	0.29
–1	–0.154	0.29	–0.106	0.29	**0.48**	0.29
0	0.022	0.29	–0.057	0.29	**0.927**	0.29
1	–0.172	0.29	–0.208	0.29	**0.506**	0.29
2	–0.101	0.29	–0.15	0.29	0.158	0.29
3	0.097	0.3	0.09	0.3	–0.007	0.3
4	0.142	0.3	0.084	0.3	–0.16	0.3
5	0.046	0.3	0.064	0.3	–0.294	0.3
6	0.146	0.31	0.18	0.31	**–0.361**	0.31
7	0.15	0.31	0.173	0.31	**–0.343**	0.31
8	0.064	0.32	0.125	0.32	–0.267	0.32
9	–0.088	0.32	–0.069	0.32	–0.146	0.32
10	–0.2	0.32	–0.181	0.32	0.027	0.32
11	–0.181	0.33	–0.205	0.33	0.214	0.33
12	–0.172	0.33	–0.243	0.33	**0.344**	0.33

*Bold text indicates statistical significance. Data on Google searches for the term “influenza” collected from Google Trends (https://trends.google.com).

Appendix Table 8. Cross-correlation coefficients between influenza cases, Google Trends, and elevated fertility rates, 2012–2018, Hyogo, Japan*

	Influenza cases vs. fertility rates		Google Trends vs. fertility rates		Influenza cases vs. Google Trends	
Lag time, mo	Correlation coefficient	Std. error	Correlation coefficient	Std. error	Correlation coefficient	Std. error
–12	–0.057	0.33	–0.006	0.33	0.327	0.33
–11	–0.054	0.33	–0.024	0.33	0.297	0.33
–10	–0.208	0.32	–0.169	0.32	0.253	0.32
–9	–0.295	0.32	–0.263	0.32	0.077	0.32
–8	–0.099	0.32	–0.13	0.32	–0.115	0.32
–7	–0.077	0.31	–0.137	0.31	–0.248	0.31
–6	0.055	0.31	–0.013	0.31	–0.308	0.31
–5	0.172	0.3	0.108	0.3	**–0.327**	0.3
–4	0.095	0.3	0.071	0.3	**–0.377**	0.3
–3	–0.003	0.3	–0.003	0.3	–0.312	0.3
–2	–0.045	0.29	–0.034	0.29	–0.126	0.29
–1	–0.059	0.29	–0.018	0.29	**0.413**	0.29
0	0.102	0.29	0.064	0.29	**0.932**	0.29
1	–0.042	0.29	–0.019	0.29	**0.545**	0.29
2	0.052	0.29	0.068	0.29	0.184	0.29
3	–0.075	0.3	–0.035	0.3	0.015	0.3
4	–0.048	0.3	–0.03	0.3	–0.12	0.3
5	0.117	0.3	0.133	0.3	–0.257	0.3
6	0.177	0.31	0.159	0.31	**–0.337**	0.31
7	0.062	0.31	0.084	0.31	**–0.32**	0.31
8	0.058	0.32	0.117	0.32	–0.25	0.32
9	–0.044	0.32	–0.035	0.32	–0.148	0.32
10	–0.153	0.32	–0.129	0.32	–0.005	0.32
11	–0.085	0.33	–0.103	0.33	0.169	0.33
12	–0.117	0.33	–0.185	0.33	0.293	0.33

*Bold text indicates statistical significance. Data on Google searches for the term "influenza" collected from Google Trends (https://trends.google.com).

Appendix Table 9. Wavelet cross-correlation coefficients between rubella cases and fertility rates, 2012–2018, Japan*

Lag time,	Tokyo, level			Kanagawa, level			Osaka, level			Hyogo, level		
mo	1	2	3	1	2	3	1	2	3	1	2	3
–12	0.031	0.006	0.013	0.192	0.004	–0.03	–0.052	–0.013	0.014	–0.066	–0.002	0.009
–11	–0.150	0.013	–0.002	–0.234	0.012	–0.052	0.061	–0.007	0.002	–0.068	–0.001	0.001
–10	0.236	0.039	–0.021	0.212	0.033	–0.064	0.077	0.023	–0.015	0.12	0.01	–0.011
–9	–0.226	0.061	–0.033	–0.132	0.059	–0.064	–0.184	0.074	–0.034	–0.17	0.029	–0.025
–8	0.100	0.020	–0.025	–0.044	0.030	–0.047	0.034	0.102	–0.040	0.092	0.030	–0.026
–7	0.043	–0.085	0.013	0.127	–0.081	–0.007	0.103	0.027	–0.001	0.041	–0.013	0.013
–6	–0.167	–0.176	0.094	–0.115	–0.185	0.074	0.019	–0.168	0.105	–0.108	–0.074	0.116
–5	0.209	–0.181	0.217	0.090	–0.178	0.197	–0.059	–0.303	0.269	0.145	–0.102	0.275
–4	–0.145	–0.081	0.357	–0.029	–0.049	0.344	–0.15	–0.167	0.444	–0.180	–0.068	0.441
–3	0.026	0.113	0.477	–0.084	0.130	0.480	0.196	0.128	0.559	0.144	–0.01	0.556
–2	0.013	0.288	0.534	0.140	0.236	0.563	0.009	0.279	0.568	–0.022	0.025	0.576
–1	0.042	0.269	0.459	–0.062	0.206	0.532	–0.132	0.241	0.442	–0.013	0.102	0.460
0	0.063	0.085	0.192	0.043	0.121	0.320	0.091	0.161	0.138	–0.12	0.265	0.169
1	–0.293	–0.048	–0.125	–0.174	0.060	0.005	–0.036	0.052	–0.185	0.184	0.308	–0.153
2	0.355	–0.033	–0.391	0.263	–0.023	–0.296	0.078	–0.117	–0.430	0.019	0.059	–0.405
3	–0.262	0.000	–0.536	–0.165	–0.141	–0.498	–0.11	–0.227	–0.532	–0.218	–0.269	–0.530
4	0.165	–0.084	–0.529	–0.002	–0.211	–0.551	0.017	–0.179	–0.470	0.212	–0.383	–0.500
5	0.008	–0.264	–0.379	0.076	–0.21	–0.454	0.037	–0.095	–0.272	–0.144	–0.271	–0.335
6	–0.280	–0.363	–0.146	–0.138	–0.207	–0.246	0.036	–0.131	–0.011	0.014	–0.095	–0.101
7	0.415	–0.275	0.103	0.242	–0.204	0.003	–0.002	–0.159	0.223	0.158	0.009	0.129
8	–0.294	–0.050	0.305	–0.215	–0.079	0.228	–0.213	0.062	0.368	–0.208	0.063	0.299
9	0.074	0.252	0.407	–0.017	0.231	0.374	0.207	0.369	0.388	0.104	0.162	0.363
10	–0.025	0.509	0.387	0.111	0.522	0.409	0.109	0.359	0.281	–0.001	0.269	0.310
11	0.093	0.507	0.264	0.036	0.497	0.333	–0.201	0.089	0.101	0.018	0.290	0.174
12	0.026	0.221	0.073	0.001	0.163	0.171	–0.005	–0.037	–0.099	–0.109	0.221	–0.010

Appendix Table 10. Wavelet cross-correlation coefficients between Google Trends and fertility rates, 2012–2018, Japan*

Lag time,	Tokyo, level			Kanagawa, level			Osaka, level			Hyogo, level		
mo	1	2	3	1	2	3	1	2	3	1	2	3
–12	0.317	0.015	0.053	0.280	0.023	0.119	0.027	–0.027	0.114	0.148	0.000	0.037
–11	–0.262	0.008	0.190	–0.236	0.026	0.168	–0.011	0.053	0.111	–0.185	0.035	0.097
–10	0.117	0.021	0.264	0.143	0.03	0.149	0.031	0.078	0.049	0.174	0.035	0.102
–9	–0.046	0.08	0.244	–0.085	0.052	0.074	0.010	0.069	–0.024	–0.187	0.029	0.058
–8	–0.082	0.081	0.143	–0.065	0.028	–0.026	–0.170	0.074	–0.072	0.044	0.034	–0.011
–7	0.234	–0.084	0.020	0.183	–0.094	–0.107	0.213	0.012	–0.070	0.125	–0.011	–0.051
–6	–0.154	–0.279	–0.055	–0.136	–0.211	–0.112	–0.069	–0.173	0.007	–0.101	–0.109	–0.012
–5	–0.110	–0.219	–0.027	–0.02	–0.168	–0.014	0.038	–0.316	0.138	–0.012	–0.119	0.107
–4	0.162	0.088	0.098	0.121	0.014	0.164	–0.155	–0.217	0.296	–0.026	–0.021	0.282
–3	–0.048	0.293	0.264	–0.149	0.169	0.380	0.110	0.082	0.457	0.142	0.008	0.458
–2	0.115	0.213	0.405	0.205	0.206	0.555	0.017	0.319	0.561	–0.016	–0.028	0.568
–1	–0.24	0.039	0.443	–0.257	0.177	0.597	0.040	0.327	0.542	–0.274	0.090	0.556
0	0.239	–0.019	0.287	0.262	0.14	0.415	–0.150	0.218	0.292	0.308	0.321	0.335
1	–0.197	0.000	0.028	–0.218	0.055	0.095	0.102	0.100	–0.045	–0.114	0.324	0.001
2	0.171	–0.002	–0.239	0.158	–0.083	–0.242	0.074	–0.078	–0.341	0.081	0.019	–0.318
3	–0.080	–0.013	–0.433	–0.062	–0.176	–0.491	–0.069	–0.245	–0.521	–0.111	–0.286	–0.529
4	–0.127	–0.048	–0.497	–0.129	–0.175	–0.581	–0.235	–0.273	–0.527	–0.030	–0.361	–0.572
5	0.318	–0.206	–0.412	0.285	–0.185	–0.496	0.451	–0.236	–0.363	0.137	–0.267	–0.44
6	–0.282	–0.378	–0.212	–0.274	–0.25	–0.279	–0.303	–0.218	–0.105	–0.122	–0.133	–0.192
7	0.095	–0.285	0.032	0.184	–0.229	–0.010	0.052	–0.128	0.159	0.129	–0.003	0.078
8	–0.042	0.085	0.253	–0.125	–0.008	0.236	–0.045	0.105	0.348	–0.211	0.110	0.293
9	0.008	0.429	0.384	–0.01	0.323	0.394	0.110	0.364	0.399	0.227	0.191	0.396
10	0.131	0.483	0.385	0.165	0.529	0.425	–0.061	0.433	0.318	–0.058	0.239	0.363
11	–0.141	0.265	0.276	–0.183	0.441	0.338	0.125	0.263	0.158	–0.146	0.277	0.233
12	0.065	0.000	0.092	0.218	0.122	0.162	–0.233	0.061	–0.032	0.167	0.254	0.044

*Data on Google searches for the term “rubella” collected from Google Trends (https://trends.google.com).

Appendix Table 11. Wavelet cross-correlation coefficients between rubella cases and Google Trends, 2012–2018, Japan

Lag time,	Tokyo			Kanagawa			Osaka			Hyogo		
mo	1	2	3	1	2	3	1	2	3	1	2	3
12	0.165	0.018	0.104	0.094	0.019	–0.025	–0.132	0.030	–0.019	0.029	0.021	0.038
–11	–0.233	0.035	0.022	–0.137	0.021	–0.008	–0.059	0.006	–0.062	–0.050	0.031	–0.003
–10	0.216	–0.001	–0.062	0.154	–0.006	–0.098	0.189	–0.006	–0.069	–0.062	0.013	–0.033
–9	–0.269	–0.144	–0.116	–0.219	–0.099	–0.069	–0.169	–0.059	–0.023	–0.008	–0.054	–0.029
–8	0.253	–0.300	–0.116	0.151	–0.224	–0.008	0.021	–0.145	0.037	0.097	–0.166	0.011
–7	–0.161	–0.256	–0.075	0.036	–0.255	0.050	0.122	–0.204	0.071	0.056	–0.242	0.065
–6	–0.117	0.104	–0.036	–0.182	–0.044	0.065	–0.055	–0.138	0.063	–0.086	–0.138	0.089
–5	0.140	0.589	–0.055	–0.067	0.387	–0.006	–0.216	0.136	–0.017	–0.224	0.217	0.037
–4	–0.074	0.767	–0.174	0.107	0.720	–0.169	0.033	0.504	–0.155	–0.032	0.625	–0.104
–3	0.569	0.373	–0.375	0.355	0.575	–0.399	0.277	0.618	–0.332	0.447	0.662	–0.322
–2	–0.624	–0.355	–0.604	–0.226	–0.068	–0.650	0.063	0.240	–0.547	0.236	0.113	–0.571
–1	0.302	–0.876	–0.805	0.283	–0.766	–0.858	0.010	–0.436	–0.762	–0.301	–0.657	–0.797
0	–0.375	–0.834	–0.909	–0.754	–0.970	–0.928	–0.166	–0.885	–0.921	–0.373	–0.980	–0.941
1	0.073	–0.306	–0.825	0.262	–0.524	–0.819	–0.412	–0.702	–0.879	–0.175	–0.582	–0.890
2	0.213	0.306	–0.640	0.058	0.196	–0.617	0.126	–0.023	–0.745	0.465	0.158	–0.729
3	–0.115	0.621	–0.434	0.157	0.659	–0.396	0.464	0.567	–0.539	–0.054	0.643	–0.516
4	0.200	0.530	–0.249	0.115	0.625	–0.201	–0.043	0.639	–0.304	0.256	0.606	–0.298
5	–0.171	0.201	–0.110	–0.197	0.259	–0.058	–0.211	0.293	–0.108	–0.252	0.229	–0.120
6	0.066	–0.102	–0.030	–0.050	–0.101	0.022	0.053	–0.086	0.003	–0.038	–0.132	–0.020
7	–0.065	–0.224	0.001	0.094	–0.250	0.050	0.014	–0.245	0.028	–0.002	–0.262	0.010
8	0.042	–0.188	0.004	0.031	–0.208	0.049	–0.091	–0.191	0.006	0.016	–0.186	0.004
9	–0.063	–0.094	–0.008	–0.085	–0.098	0.035	0.059	–0.068	–0.018	0.092	–0.053	–0.011
10	0.013	–0.012	–0.022	–0.043	–0.010	0.013	0.132	0.013	–0.028	–0.099	0.030	–0.021
11	0.044	0.028	–0.029	0.061	0.029	–0.012	–0.242	0.033	–0.027	0.018	0.043	–0.022
12	–0.025	0.033	–0.030	–0.039	0.035	–0.038	0.010	0.023	–0.023	–0.060	0.020	–0.023

*Data on Google searches for the term “rubella” collected from Google Trends (https://trends.google.com).

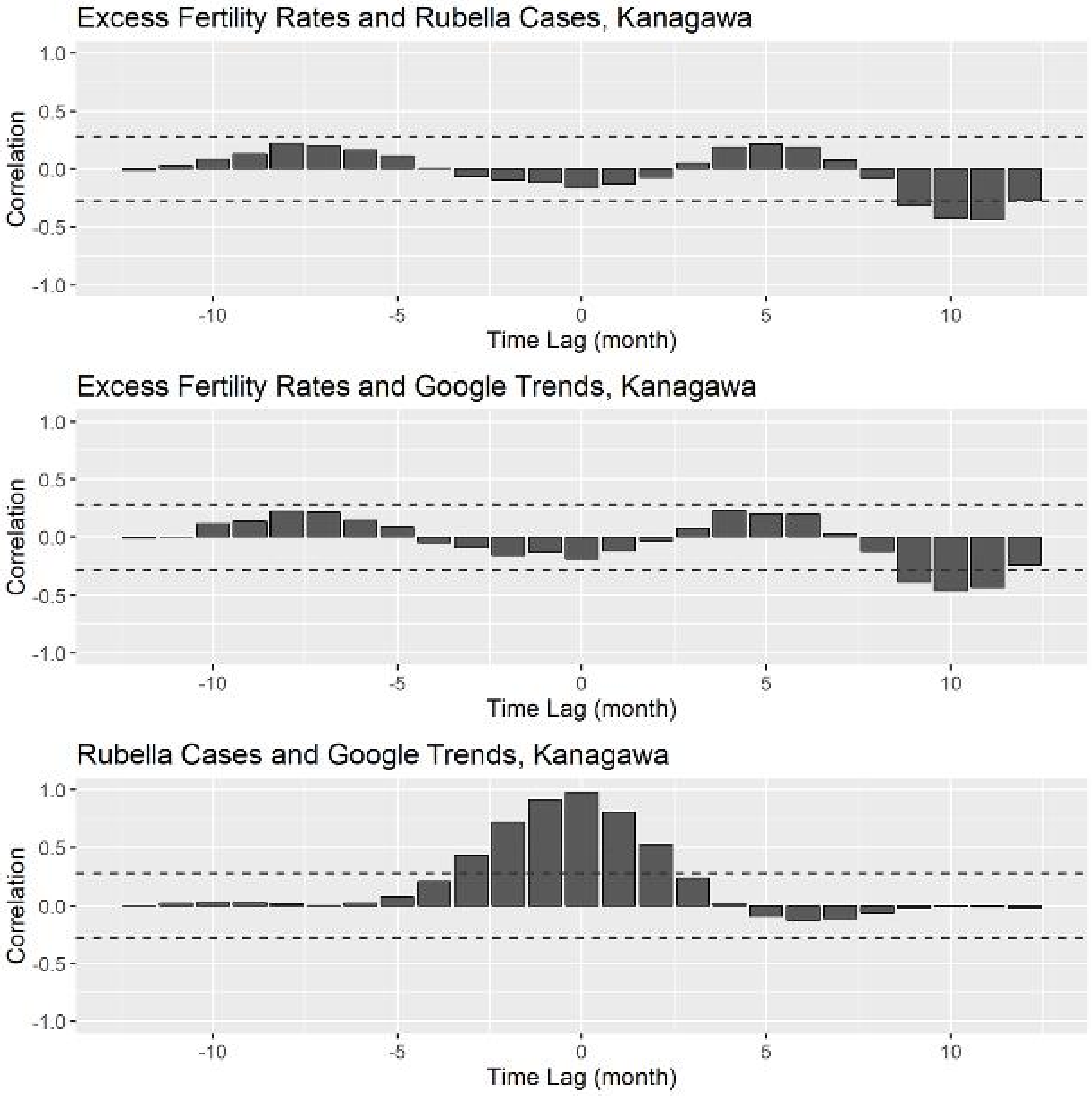

Appendix Figure 1. Cross-correlation between rubella cases, Google Trends, and elevated fertility rates, 2012–2018, Kanagawa, Japan. Cross-correlation coefficients were calculated in each lag (–12 months) and lead period (+12 months) including 0. Upper limit, 0.28; lower limit, –0.28. Bars represent the cross-correlation coefficients in time series between (top panel) fertility rates and rubella cases; (middle panel) fertility rate and Google searches for the term “rubella”; and (bottom panel) rubella cases and Google searches for “rubella.” Horizontal dashed lines represent confidence limits for the null hypothesis of 0 true cross-correlation coefficients between the 2 time-series. Data on Google searches aggregated from Google Trends (https://trends.google.com).

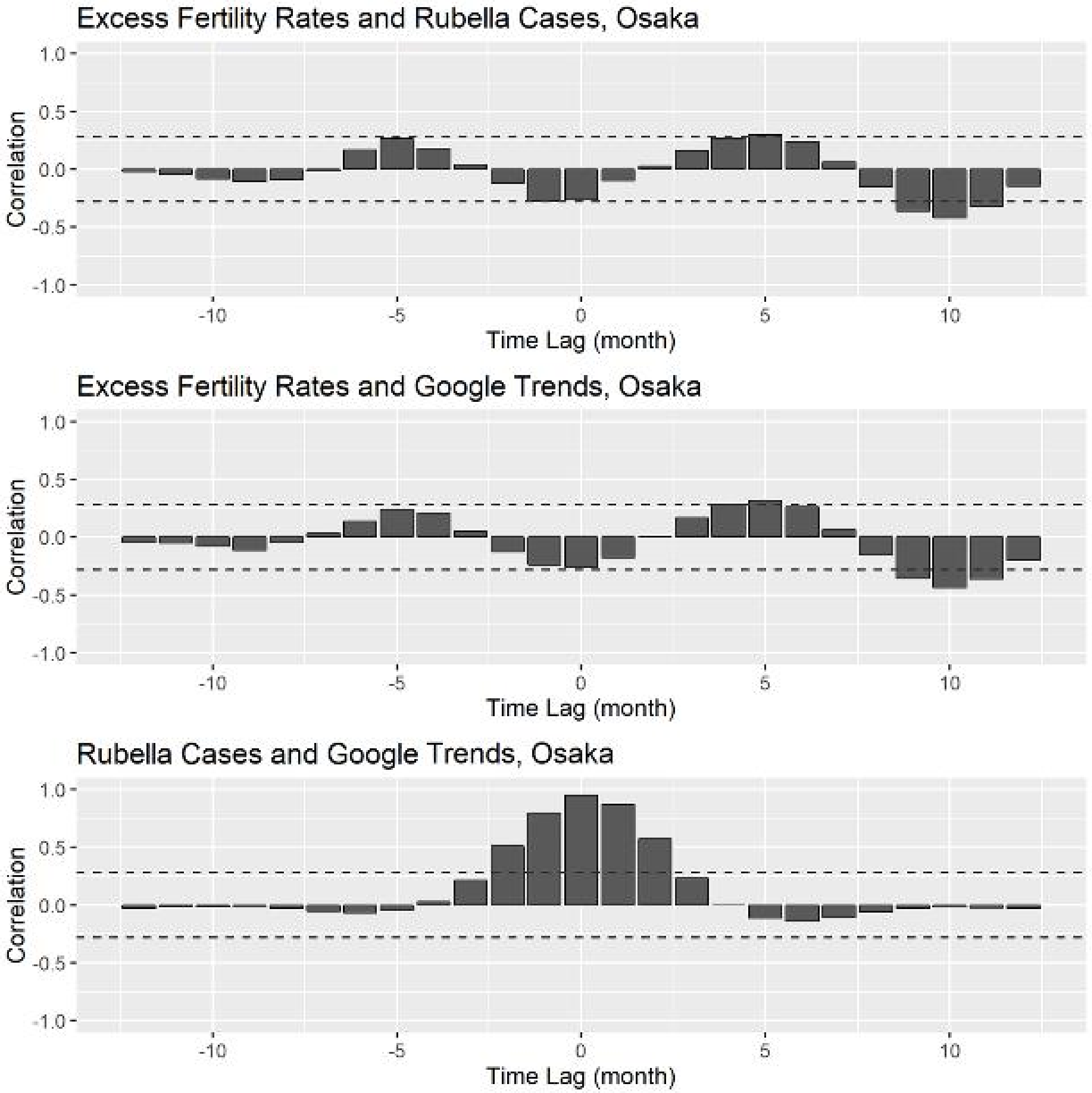

Appendix Figure 2. Cross-correlation between rubella cases, Google Trends, and elevated fertility rates, 2012–2018, Osaka, Japan. Cross-correlation coefficients were calculated in each lag (–12 months) and lead period (+12 months) including 0. Cross-correlation coefficients were calculated in each lag (–12 months) and lead period (+12 months) including 0. Upper limit, 0.28; lower limit, –0.28. Bars represent the cross-correlation coefficients in time series between (top panel) fertility rates and rubella cases; (middle planel) fertility rate and Google searches for the term “rubella”; and (bottom panel) rubella cases and Google searches for “rubella.” Horizontal dashed lines represent confidence limits for the null hypothesis of 0 true cross-correlation coefficients between the 2 time-series. Data on Google searches aggregated from Google Trends (https://trends.google.com).

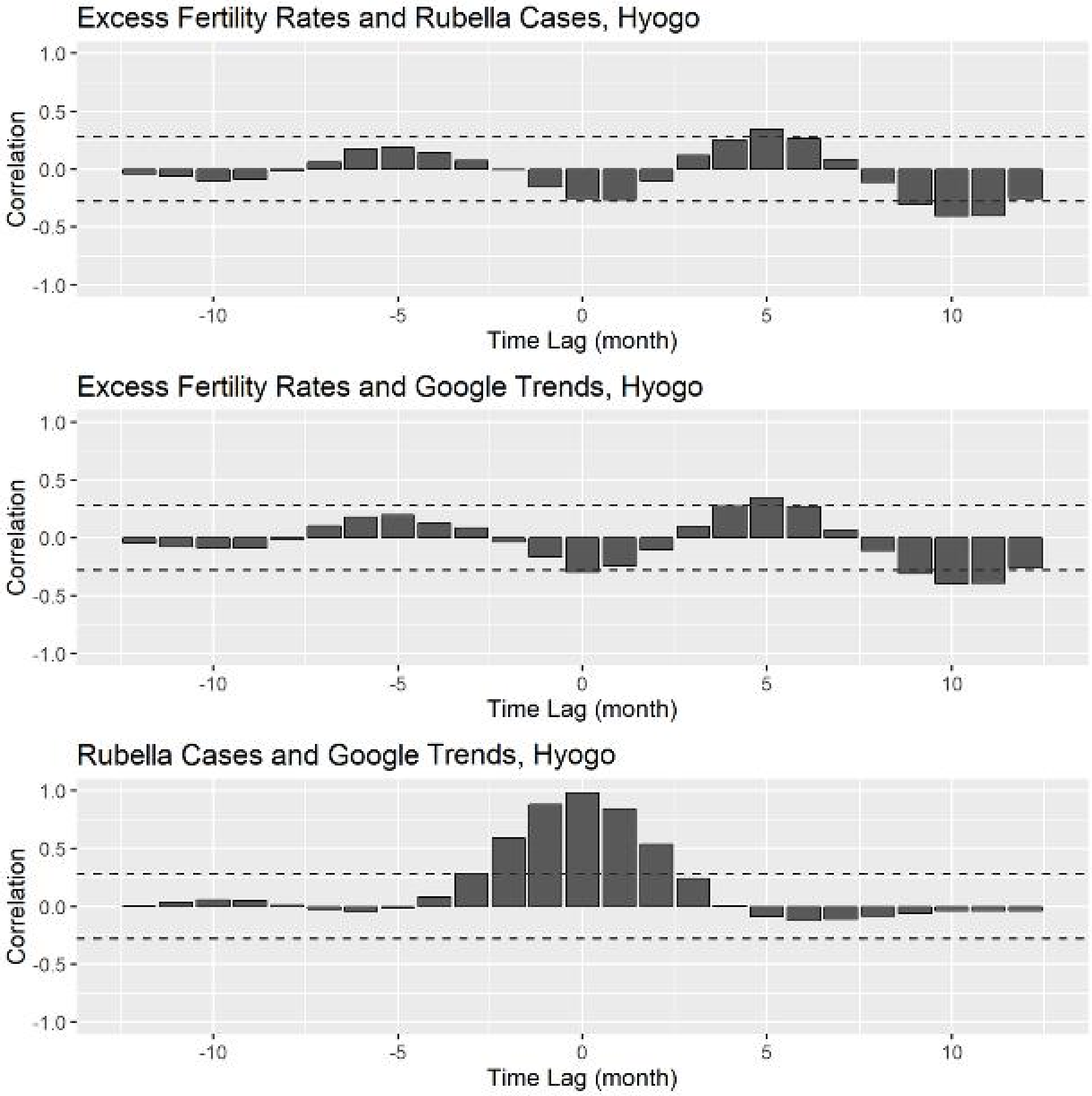

Appendix Figure 3. Cross-correlation between rubella cases, Google Trends, and elevated fertility rates, 2012–2018, Hyogo, Japan. Cross-correlation coefficients were calculated in each lag (–12 months) and lead period (+12 months) including 0. Cross-correlation coefficients were calculated in each lag (–12 months) and lead period (+12 months) including 0. Upper limit, 0.28; lower limit, –0.28. Bars represent the cross-correlation coefficients in time series between (top panel) fertility rates and rubella cases; (middle planel) fertility rate and Google searches for the term “rubella”; and (bottom panel) rubella cases and Google searches for “rubella.” Horizontal dashed lines represent confidence limits for the null hypothesis of 0 true cross-correlation coefficients between the 2 time-series. Data on Google searches aggregated from Google Trends (https://trends.google.com).

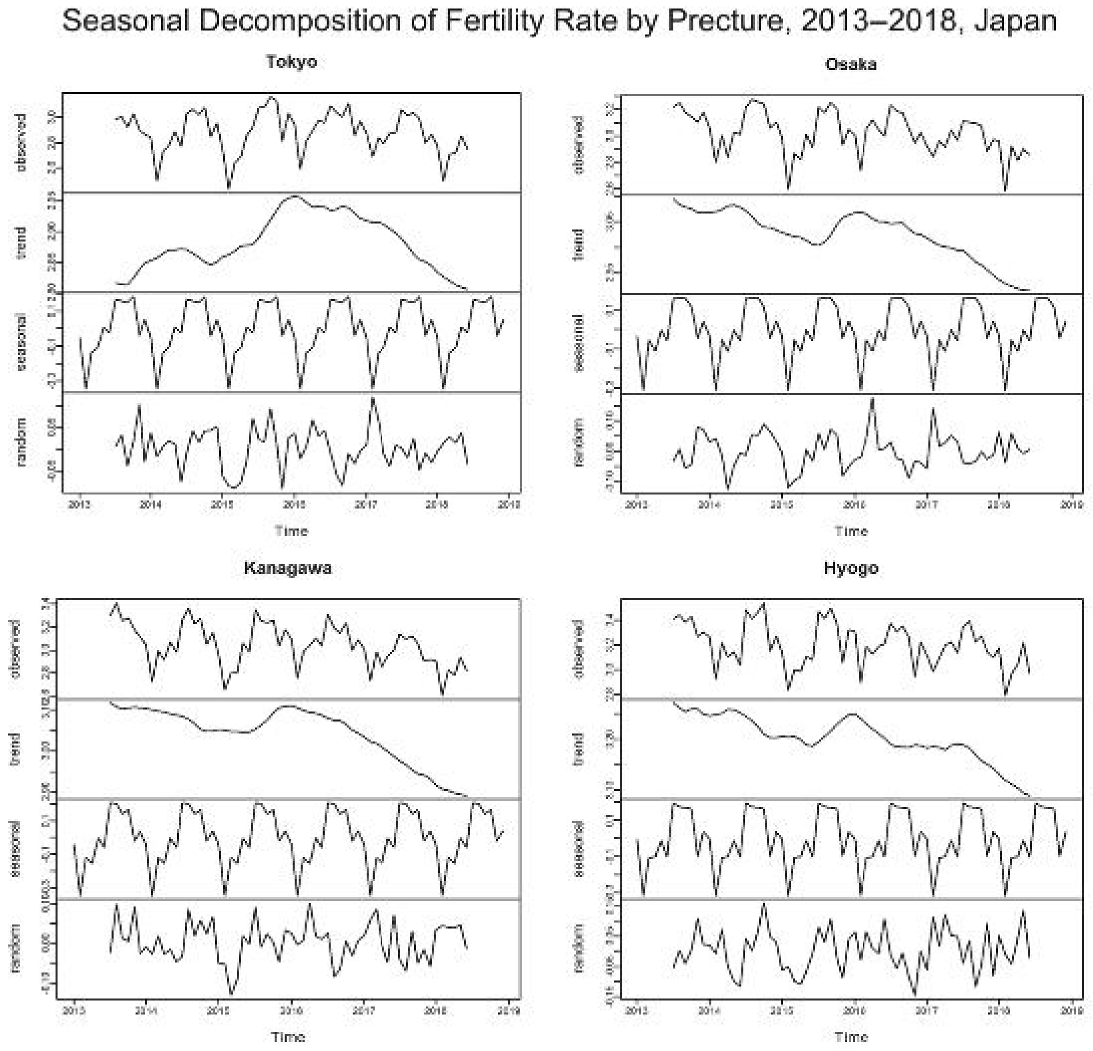

Appendix Figure 4. Decomposition of additive time series for fertility rates by prefecture, 2013–2018, Japan. A) Tokyo; B) Kanagawa; C) Osaka; and D) Hyogo. Observed data, trend component, seasonal component and random component are displayed.

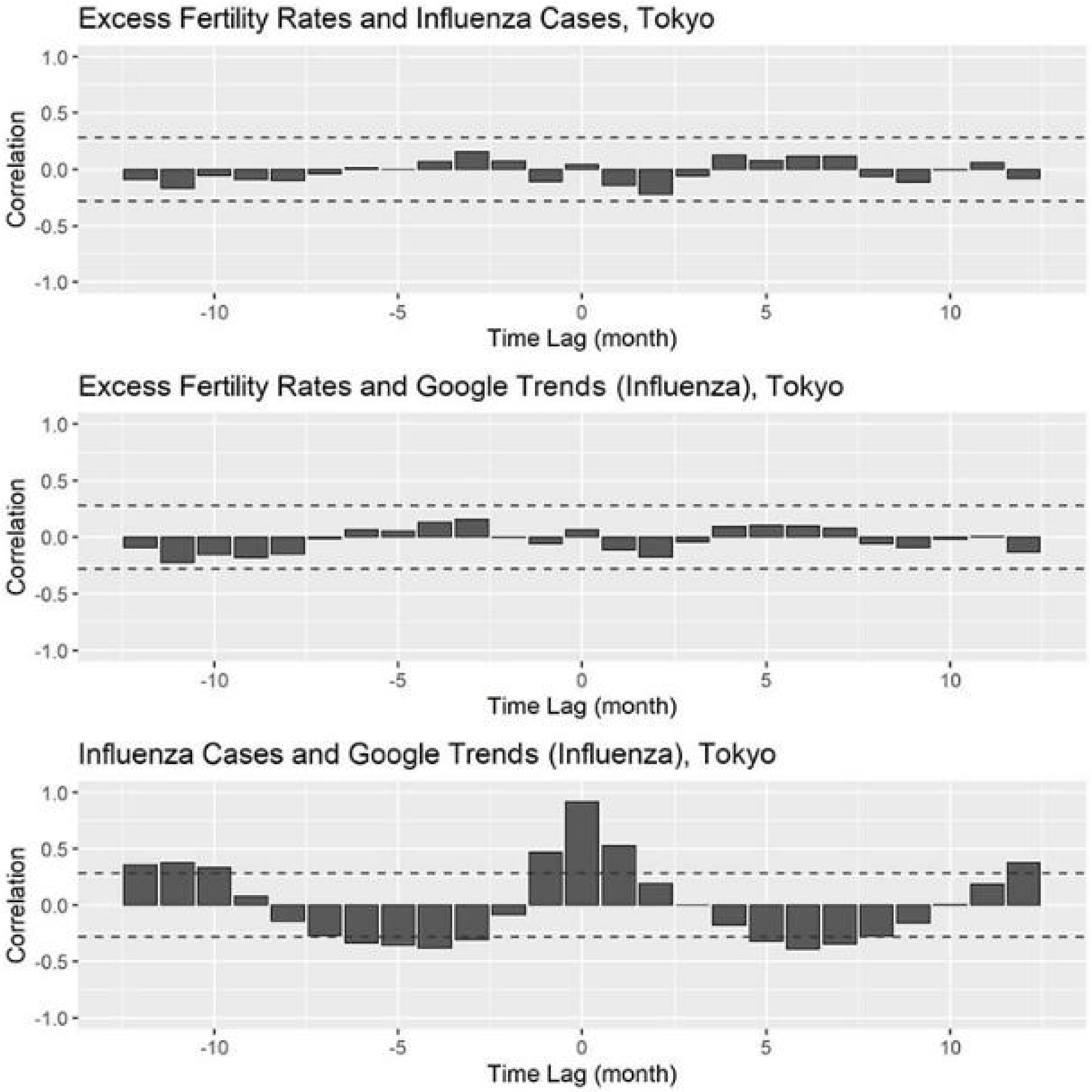

Appendix Figure 5. Cross-correlation between influenza cases, Google Trends, and elevated fertility rates, 2012–2018, Tokyo, Japan. Cross-correlation coefficients were calculated in each lag (–12 months) and lead period (+12 months) including 0. Cross-correlation coefficients were calculated in each lag (–12 months) and lead period (+12 months) including 0. Upper limit, 0.28; lower limit, –0.28. Bars represent the cross-correlation coefficients in time series between (top panel) fertility rates and influenza cases; (middle planel) fertility rate and Google searches for the term "influenza"; and (bottom panel) influenza cases and Google searches for "influenza." Horizontal dashed lines represent confidence limits for the null hypothesis of 0 true cross-correlation coefficients between the 2 time-series. Data on Google searches aggregated from Google Trends (https://trends.google.com).

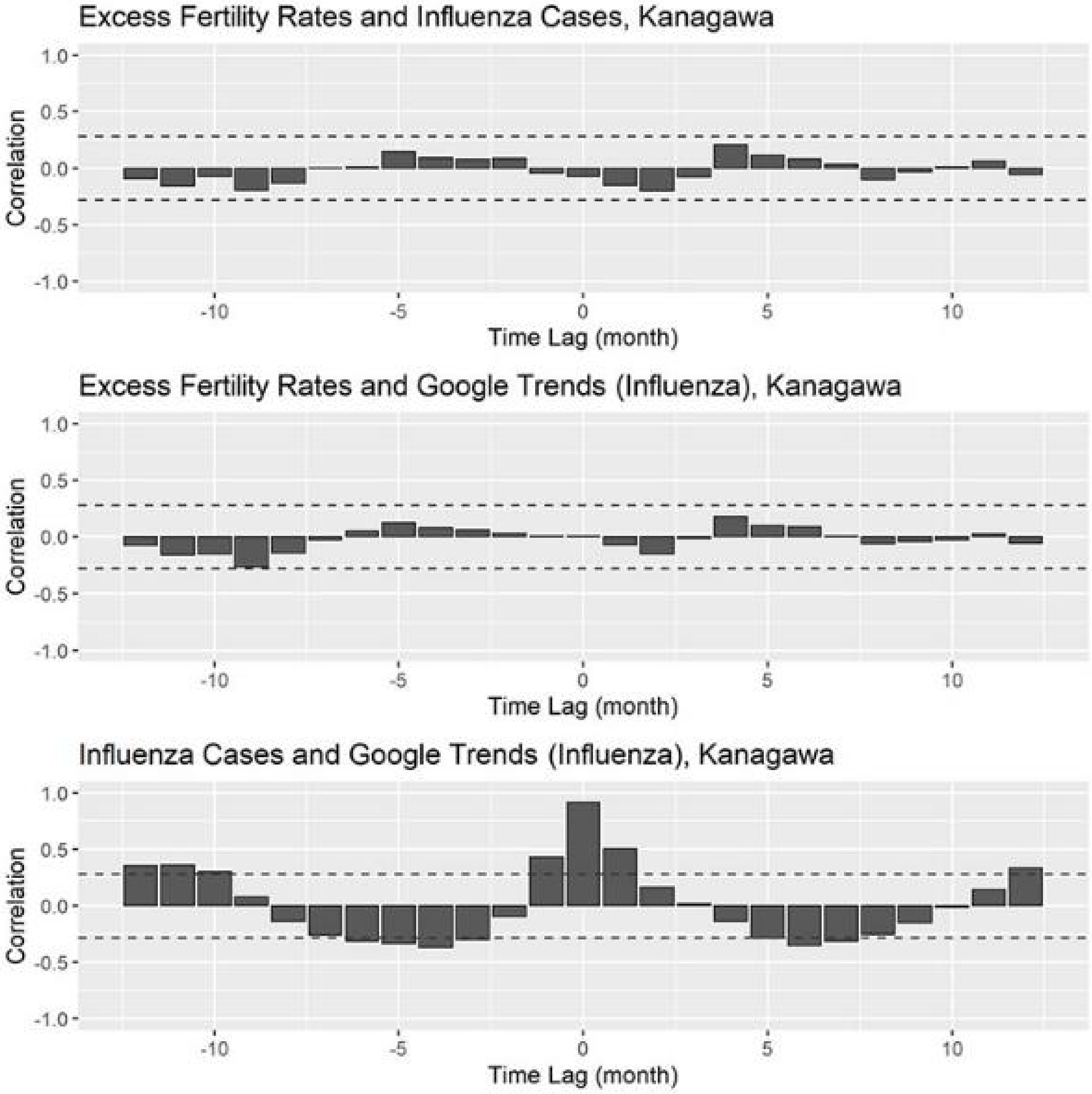

Appendix Figure 6. Cross-correlation between influenza cases, Google Trends, and elevated fertility rates, 2012–2018, Kanagawa, Japan. Cross-correlation coefficients were calculated in each lag (–12 months) and lead period (+12 months) including 0. Cross-correlation coefficients were calculated in each lag (–12 months) and lead period (+12 months) including 0. Upper limit, 0.28; lower limit, –0.28. Bars represent the cross-correlation coefficients in time series between (top panel) fertility rates and influenza cases; (middle planel) fertility rate and Google searches for the term "influenza"; and (bottom panel) influenza cases and Google searches for "influenza." Horizontal dashed lines represent confidence limits for the null hypothesis of 0 true cross-correlation coefficients between the 2 time-series. Data on Google searches aggregated from Google Trends (https://trends.google.com).

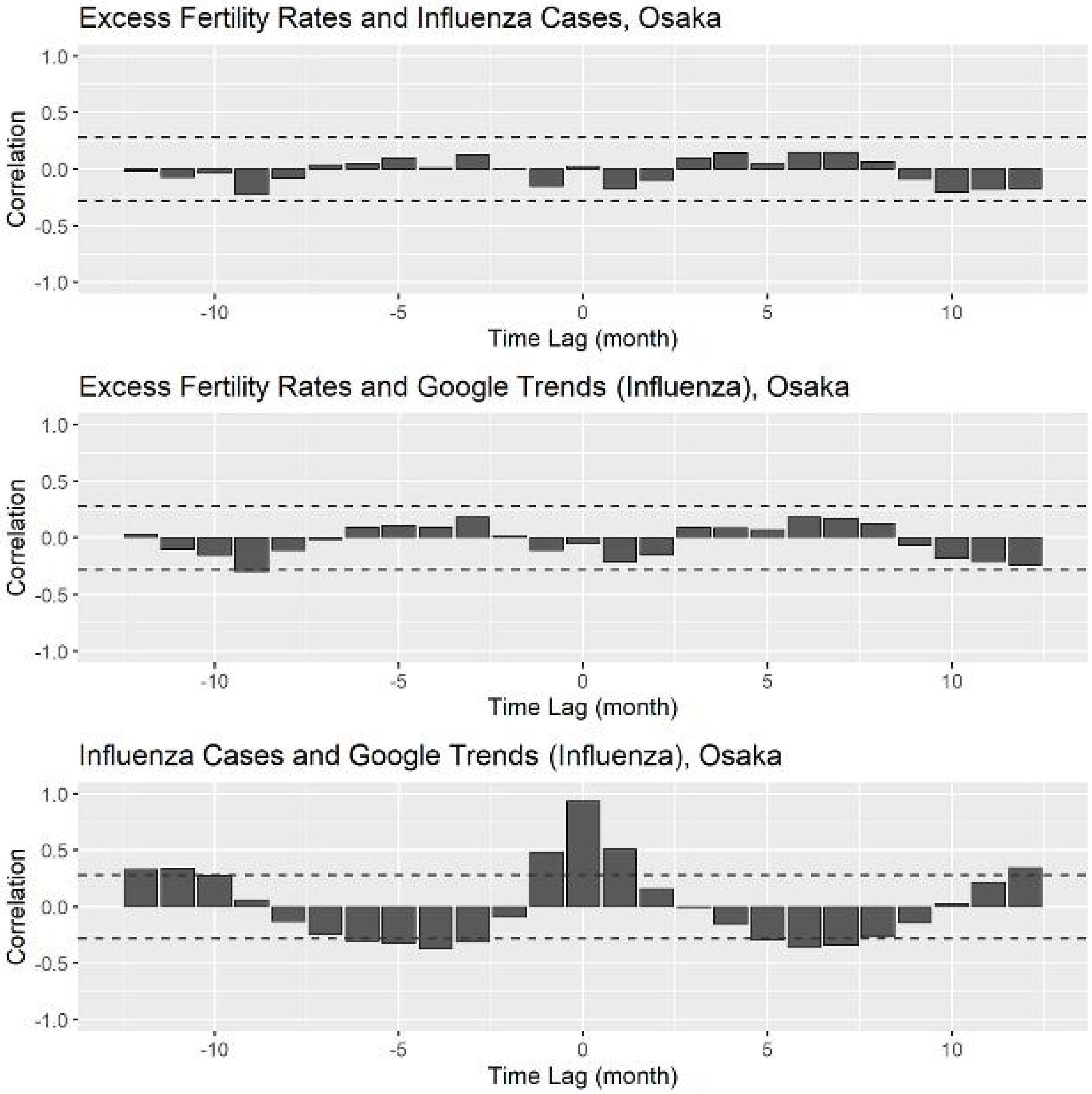

Appendix Figure 7. Cross-correlation between influenza cases, Google Trends, and elevated fertility rates, 2012–2018, Osaka, Japan. Cross-correlation coefficients were calculated in each lag (–12 months) and lead period (+12 months) including 0. Cross-correlation coefficients were calculated in each lag (–12 months) and lead period (+12 months) including 0. Upper limit, 0.28; lower limit, –0.28. Bars represent the cross-correlation coefficients in time series between (top panel) fertility rates and influenza cases; (middle planel) fertility rate and Google searches for the term “influenza”; and (bottom panel) influenza cases and Google searches for “influenza.” Horizontal dashed lines represent confidence limits for the null hypothesis of 0 true cross-correlation coefficients between the 2 time-series. Data on Google searches aggregated from Google Trends (https://trends.google.com).

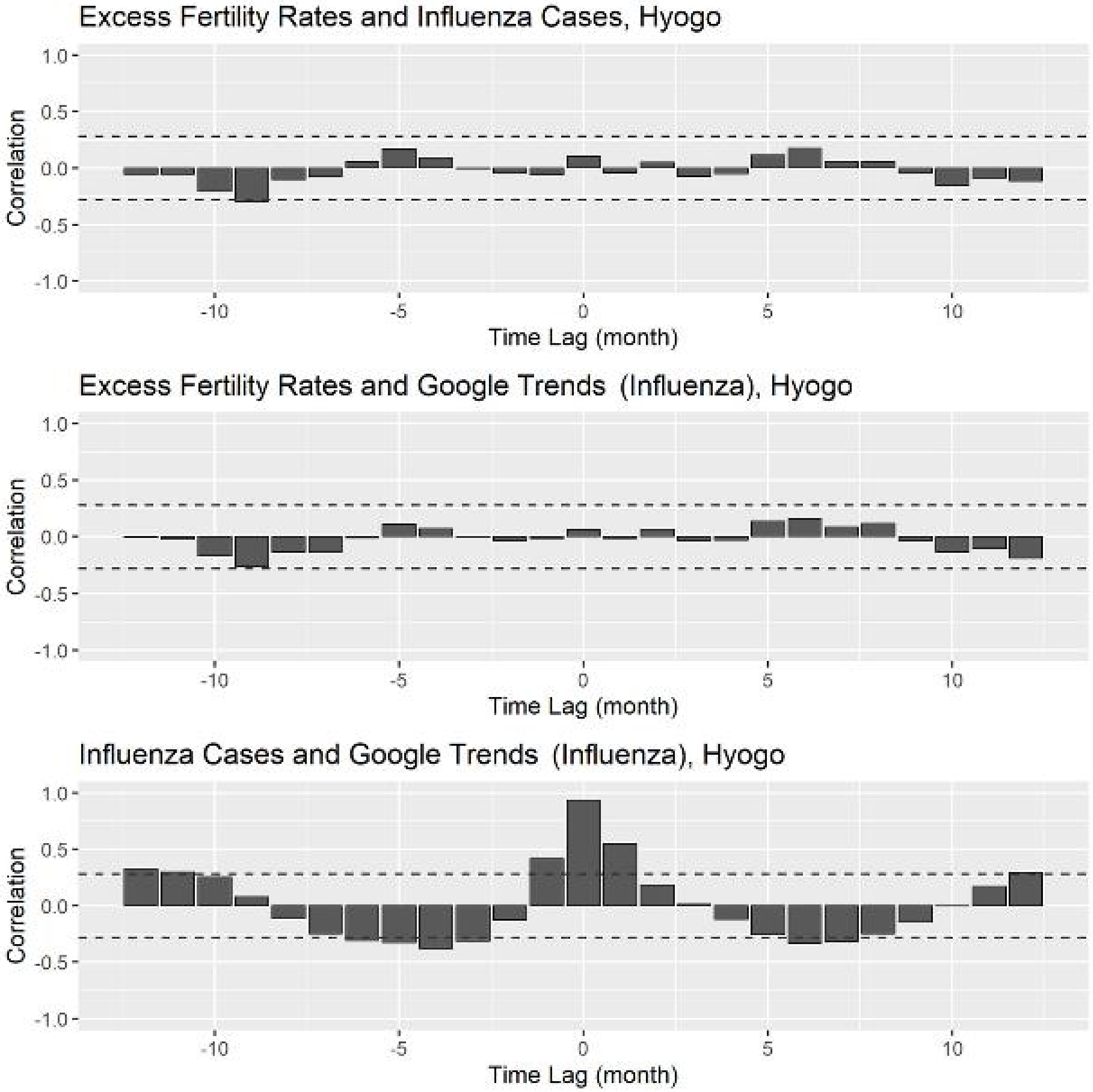

Appendix Figure 8. Cross-correlation between influenza cases, Google searches for the term "influenza," and elevated fertility rates, 2012–2018, Hyogo, Japan. Cross-correlation coefficients were calculated in each lag (–12 months) and lead period (+12 months) including 0. Cross-correlation coefficients were calculated in each lag (–12 months) and lead period (+12 months) including 0. Upper limit, 0.28; lower limit, –0.28. Bars represent the cross-correlation coefficients in time series between (top panel) fertility rates and influenza cases; (middle planel) fertility rate and Google searches for the term "influenza"; and (bottom panel) influenza cases and Google searches for "influenza." Horizontal dashed lines represent confidence limits for the null hypothesis of 0 true cross-correlation coefficients between the 2 time-series. Data on Google searches aggregated from Google Trends (https://trends.google.com).

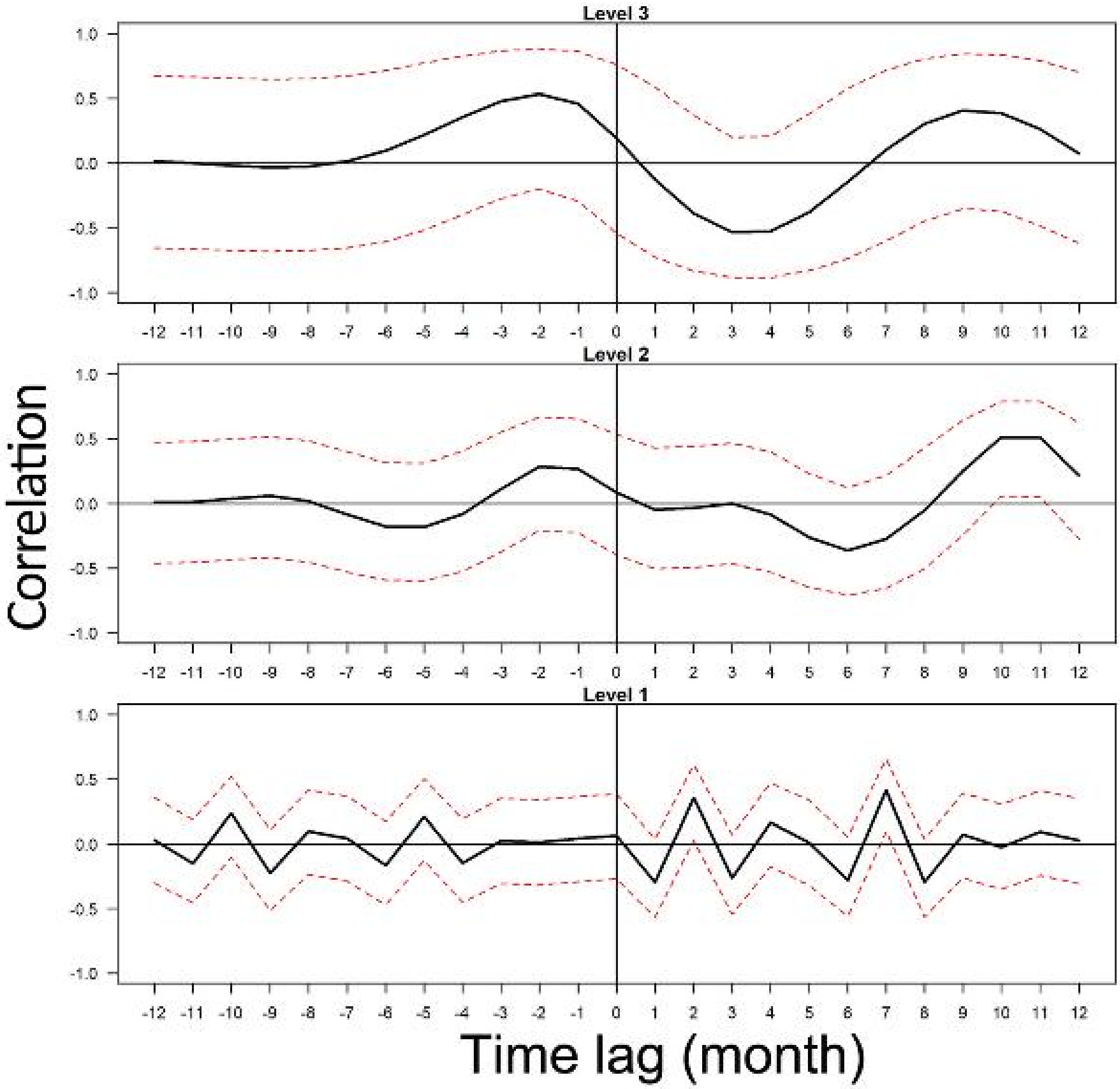

Appendix Figure 9. Wavelet cross-correlation between rubella cases and fertility rates, 2012–2018, Tokyo, Japan. Dotted line represents 95% confidence interval.

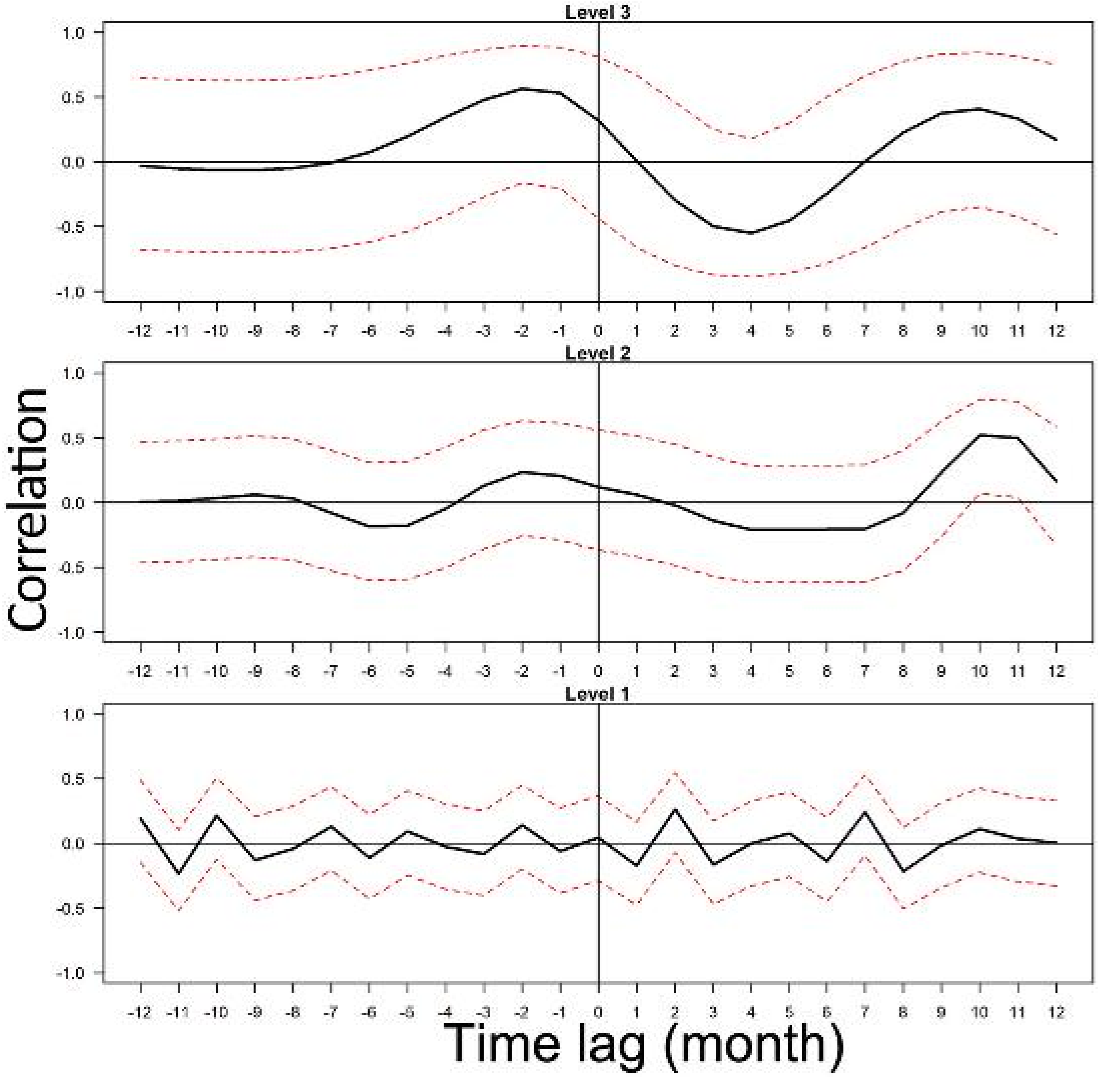

Appendix Figure 10. Wavelet cross-correlation between rubella cases and fertility rates, 2012–2018, Kanagawa, Japan. Dotted line represents 95% confidence interval.

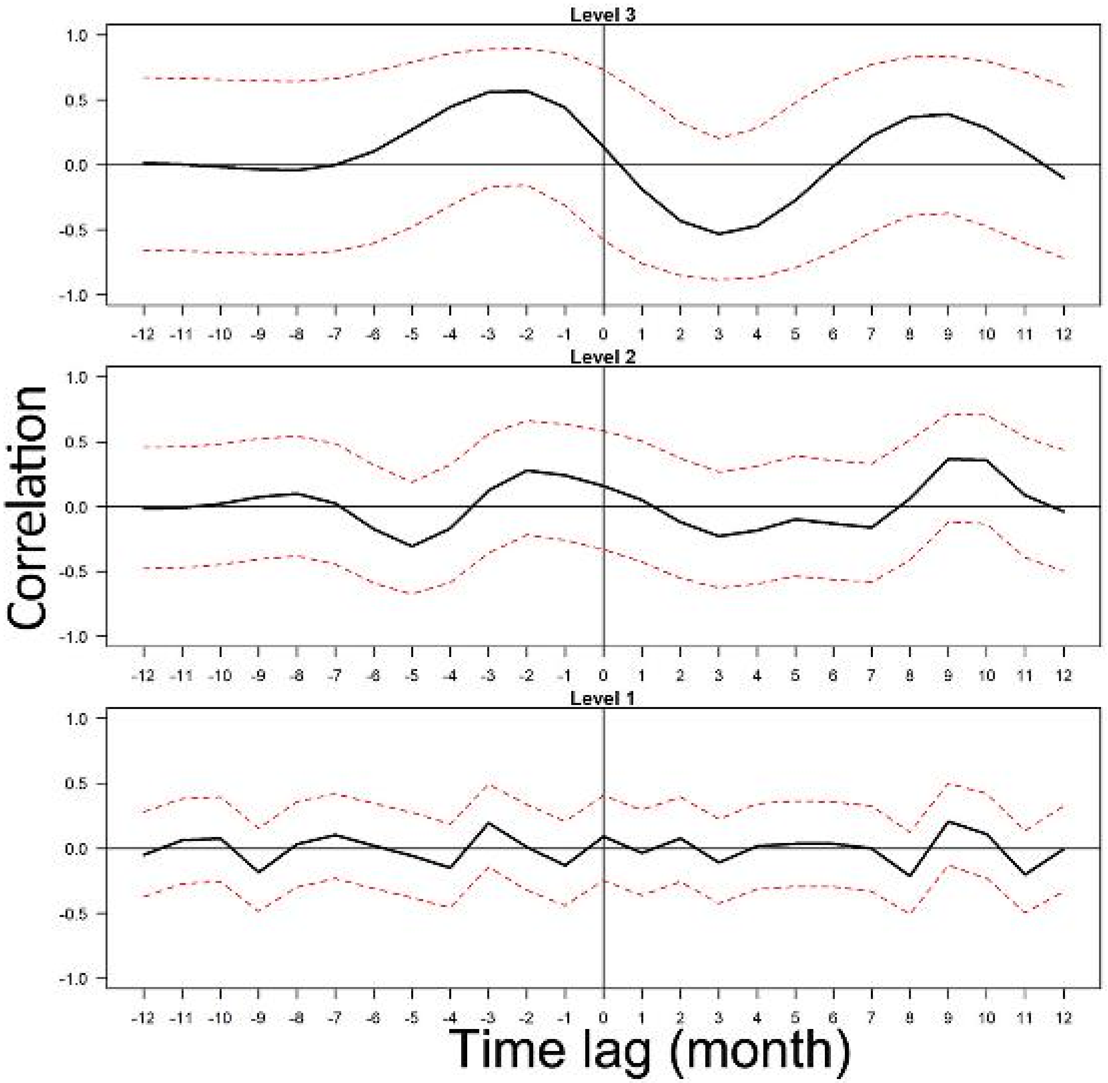

Appendix Figure 11. Wavelet cross-correlation between rubella cases and fertility rates, 2012–2018, Osaka, Japan. Dotted line represents 95% confidence interval.

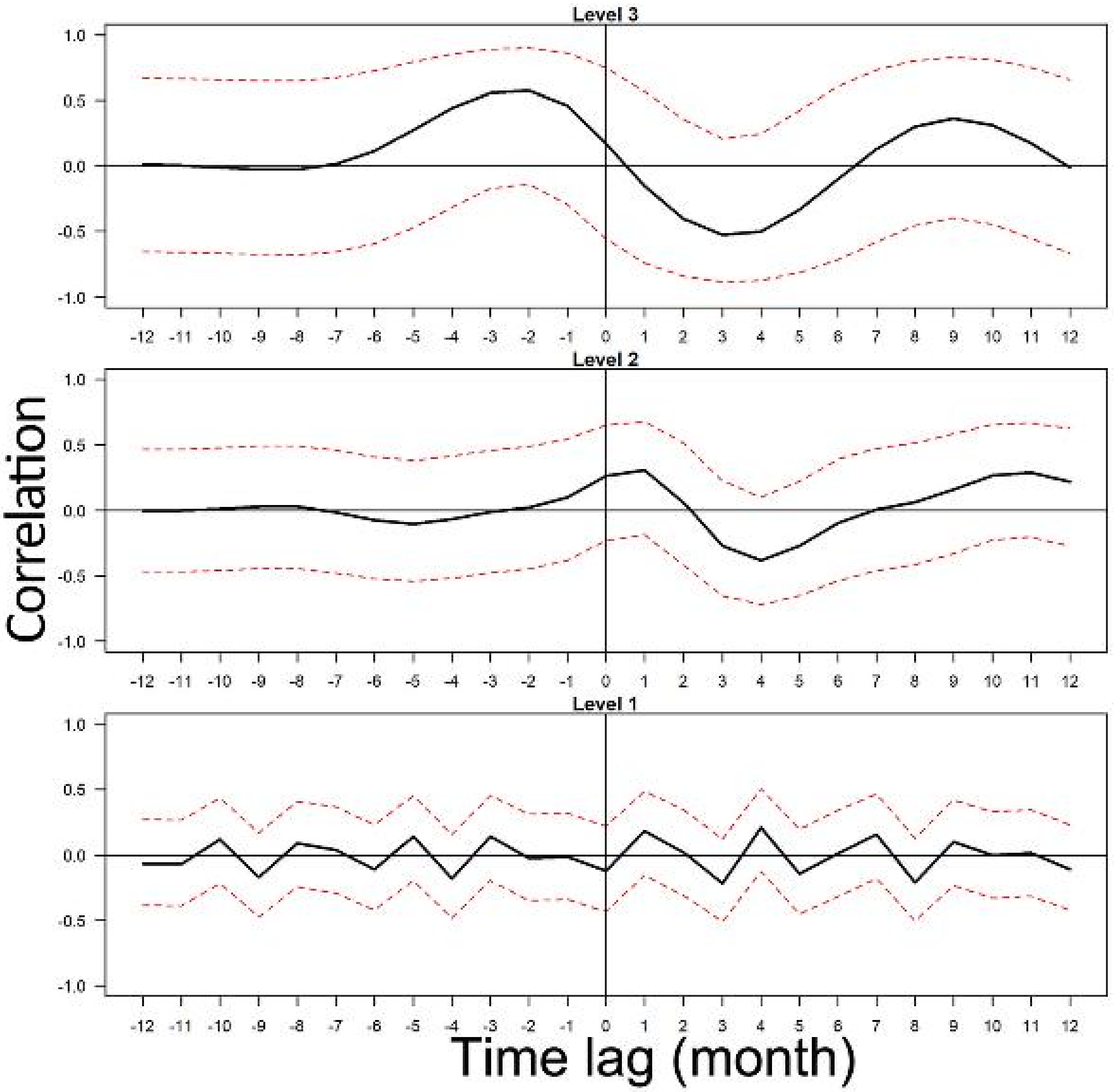

Appendix Figure 12. Wavelet cross-correlation between rubella cases and fertility rates, 2012–2018, Hyogo, Japan. Dotted line represents 95% confidence interval.

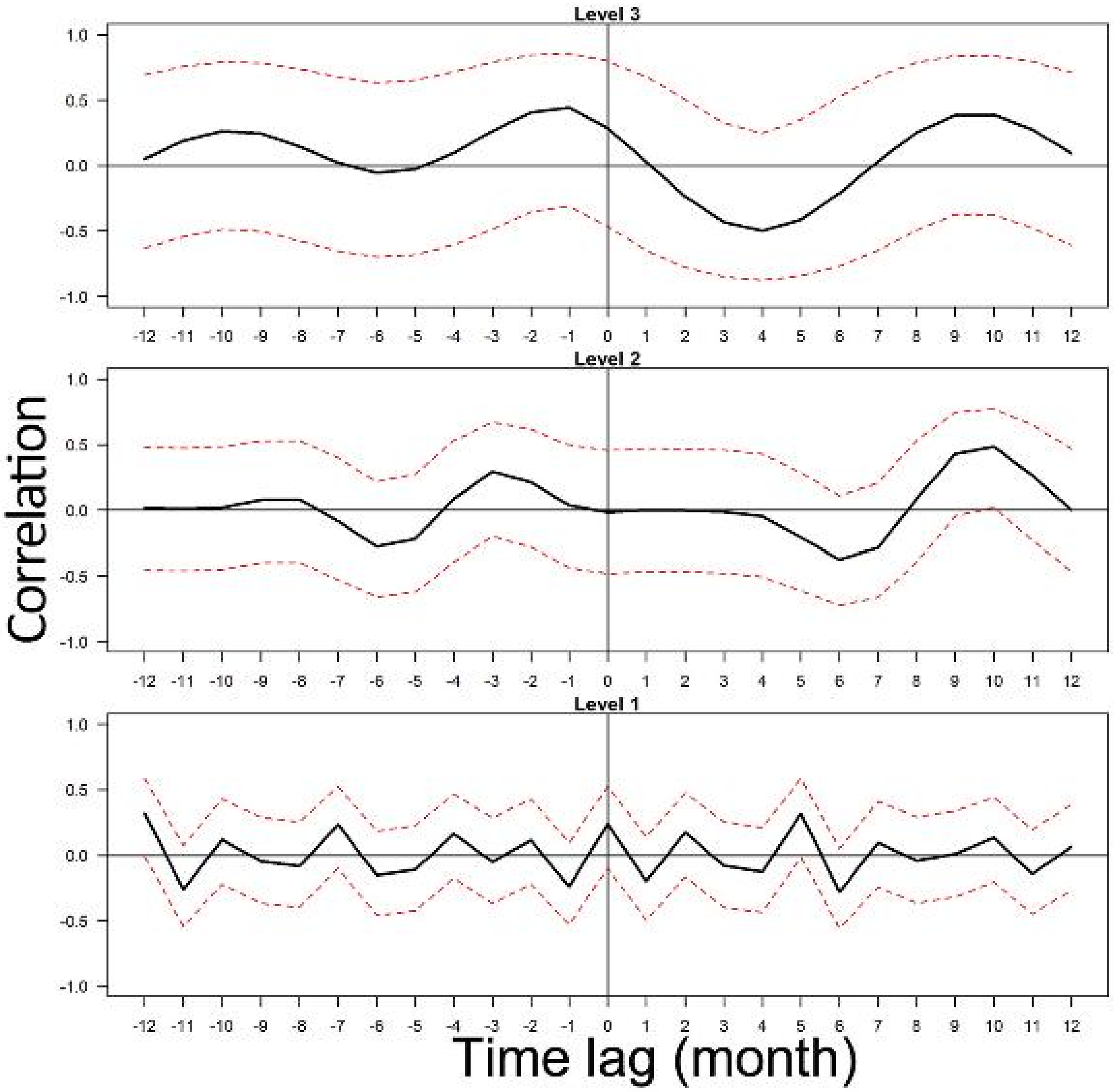

Appendix Figure 13. Wavelet cross-correlation between Google Trends and fertility rates, 2012–2018, Tokyo, Japan. Dotted line represents 95% confidence interval.

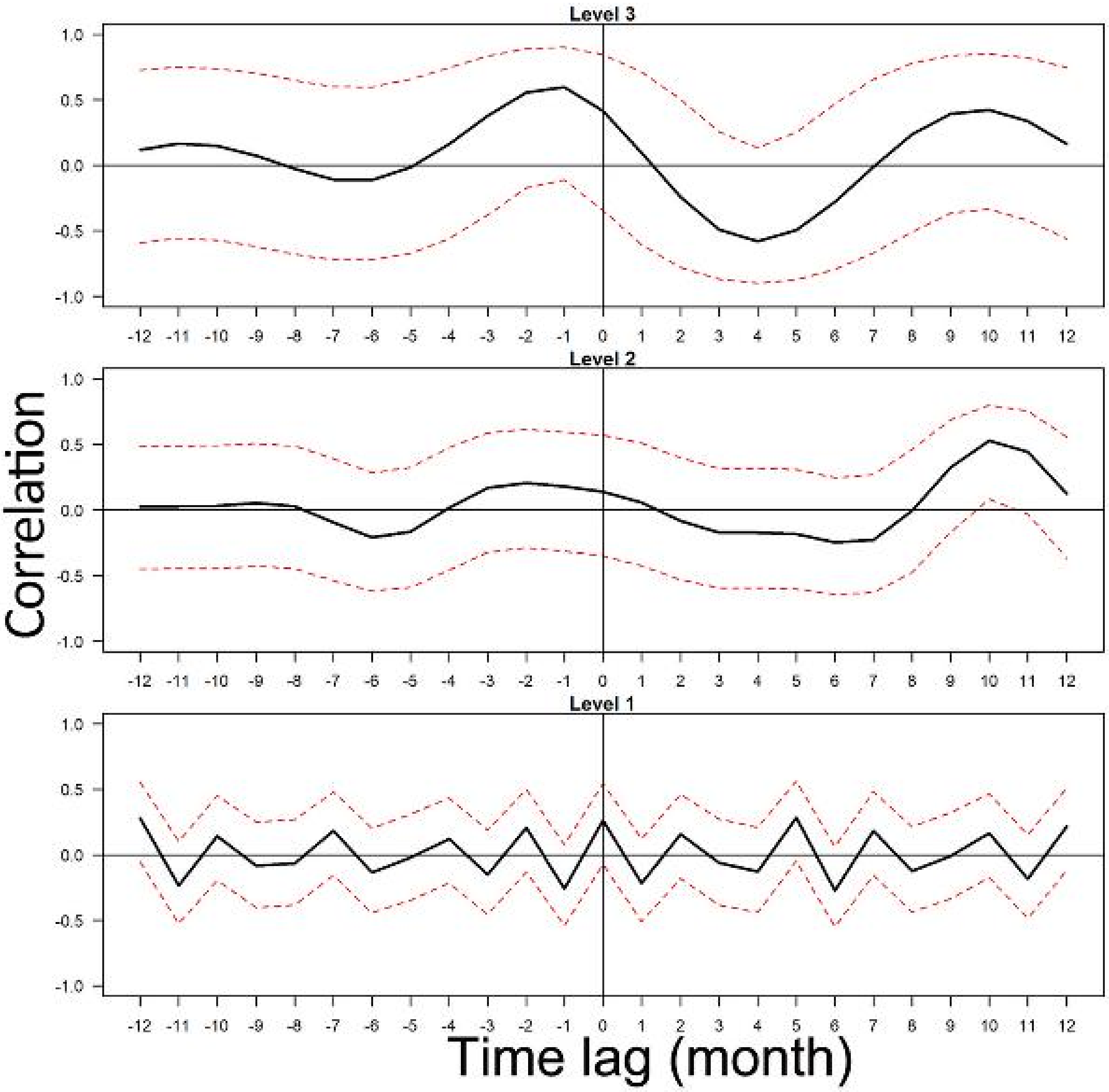

Appendix Figure 14. Wavelet cross-correlation between Google searches for “rubella” and fertility rates, 2012–2018, Kanagawa, Japan. Dotted line represents 95% confidence interval.

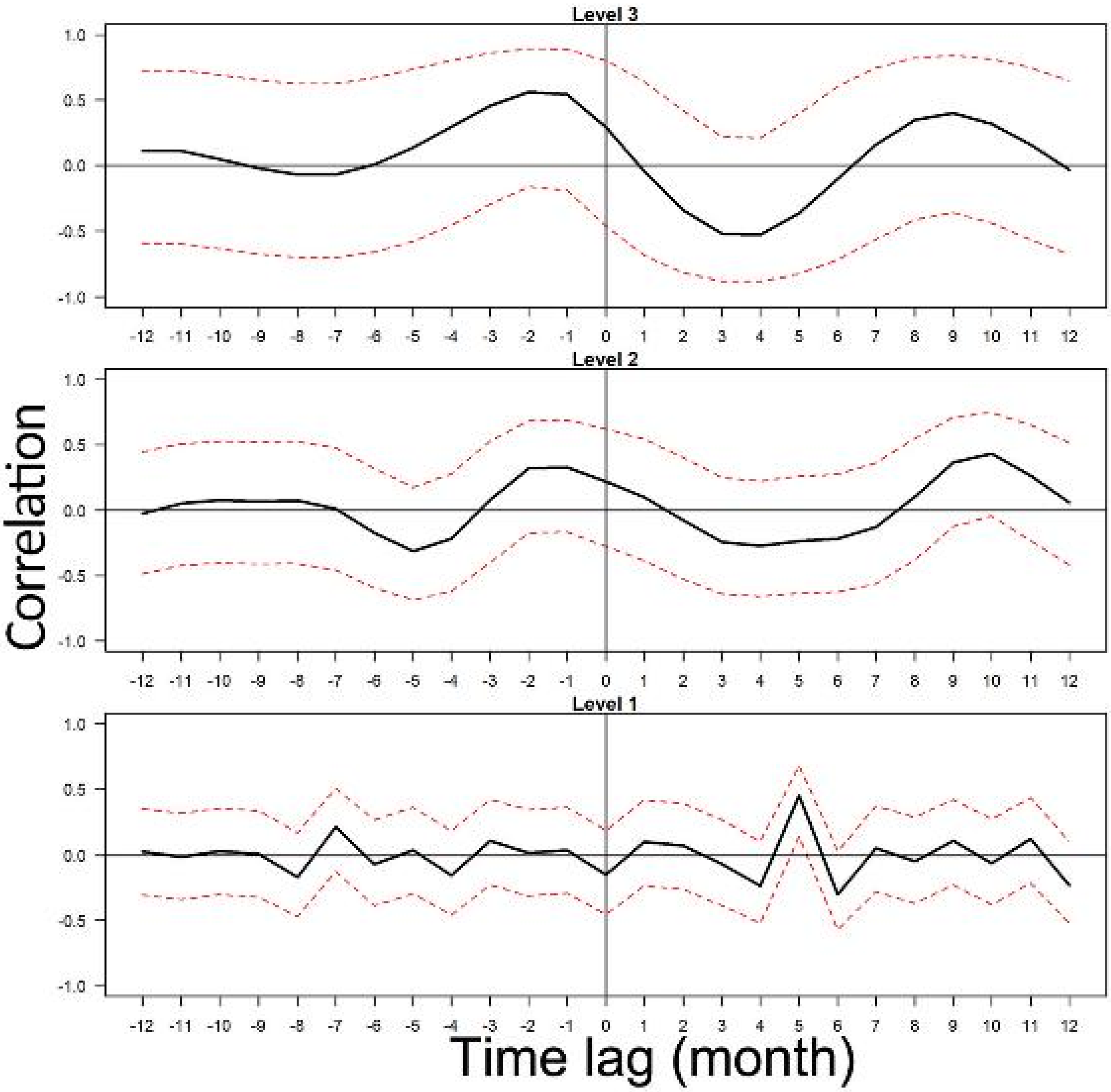

Appendix Figure 15. Wavelet cross-correlation between Google searches for “rubella” and fertility rates, 2012–2018, Osaka, Japan. Dotted line represents 95% confidence interval.

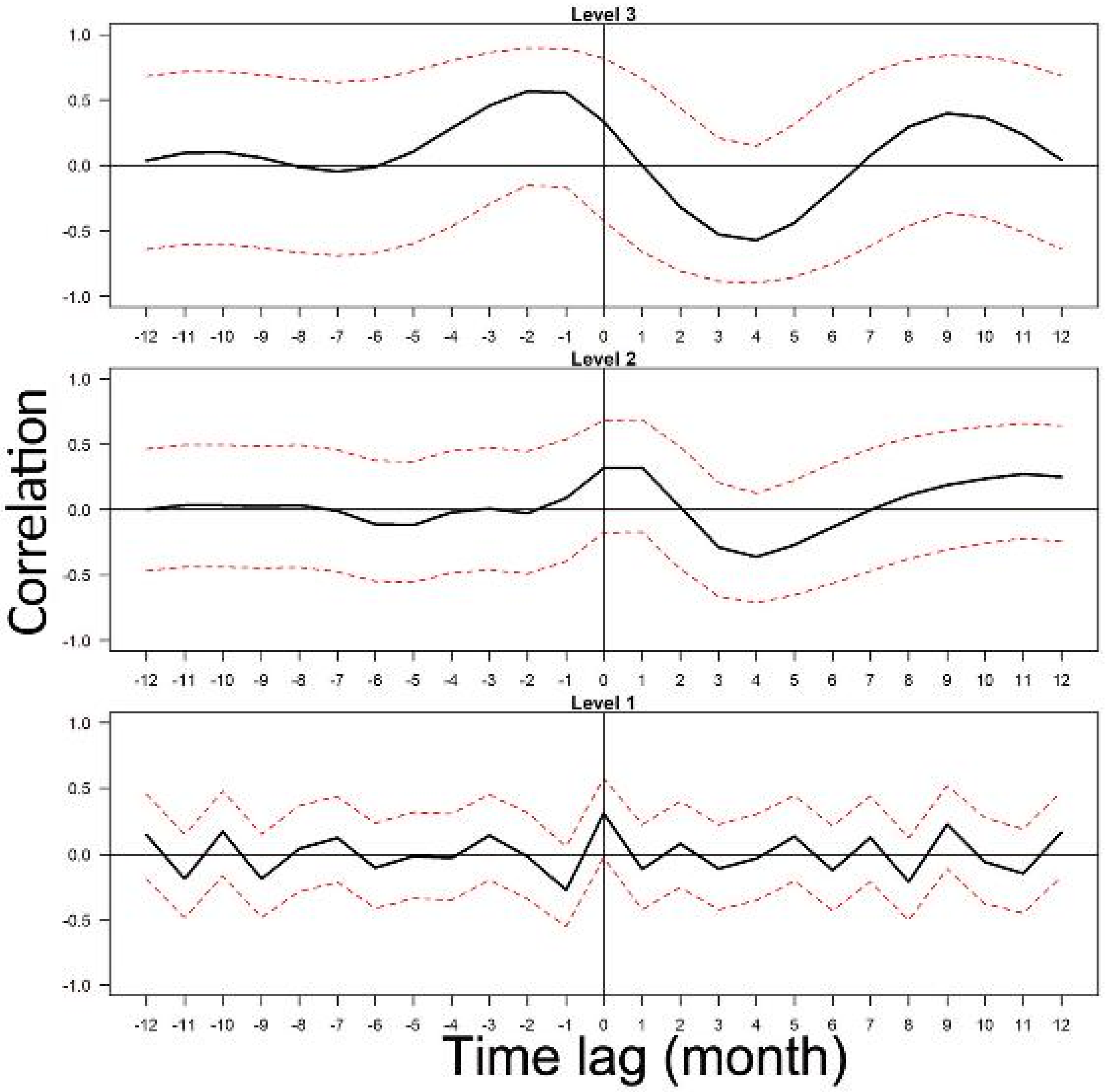

Appendix Figure 16. Wavelet cross-correlation between Google Trends and fertility rates, 2012–2018, Hyogo, Japan. Dotted line represents 95% confidence interval.

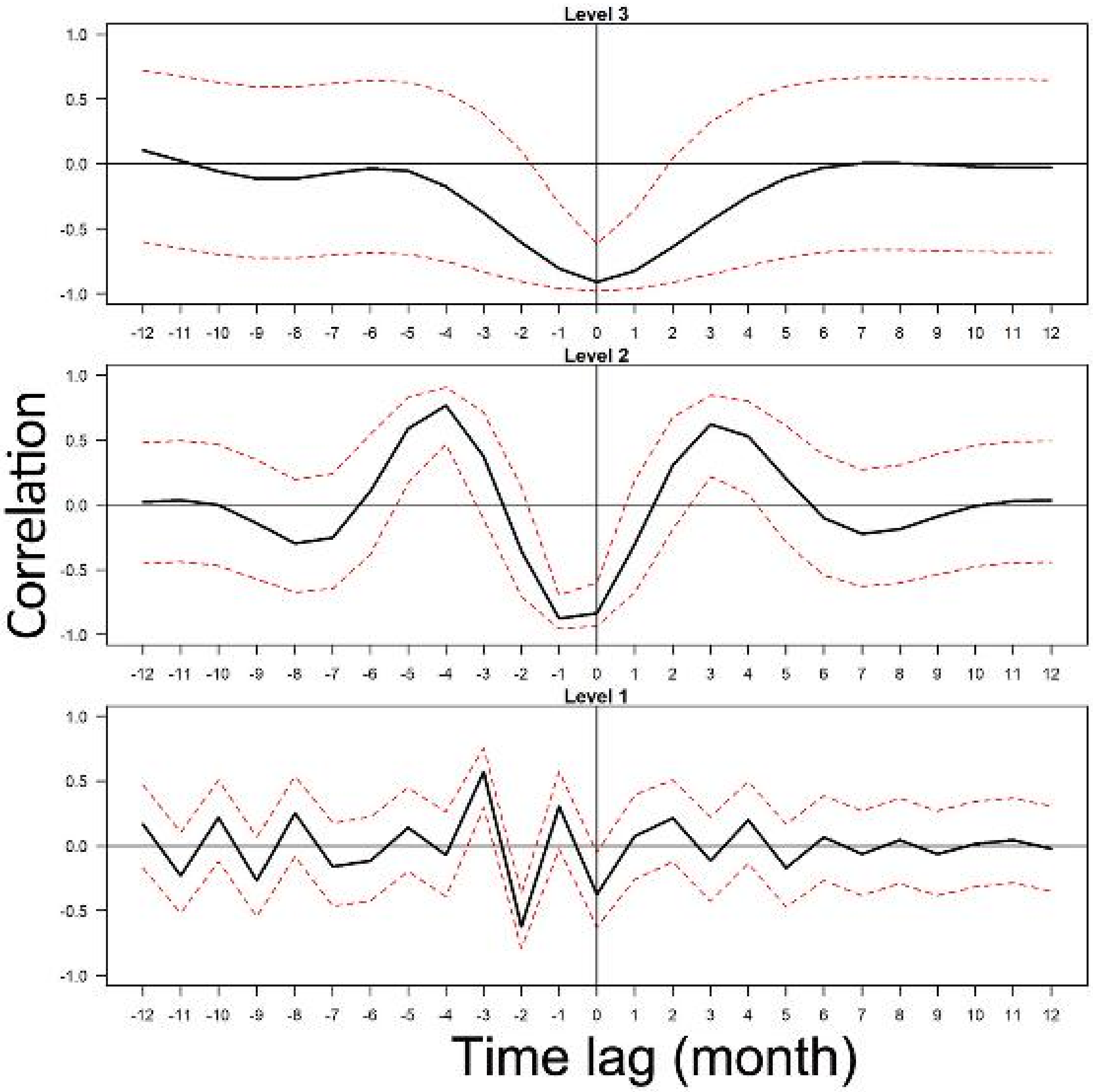

Appendix Figure 17. Wavelet cross-correlation between rubella cases and Google searches for “rubella,” 2012–2018, Tokyo, Japan. Dotted line represents 95% confidence interval.

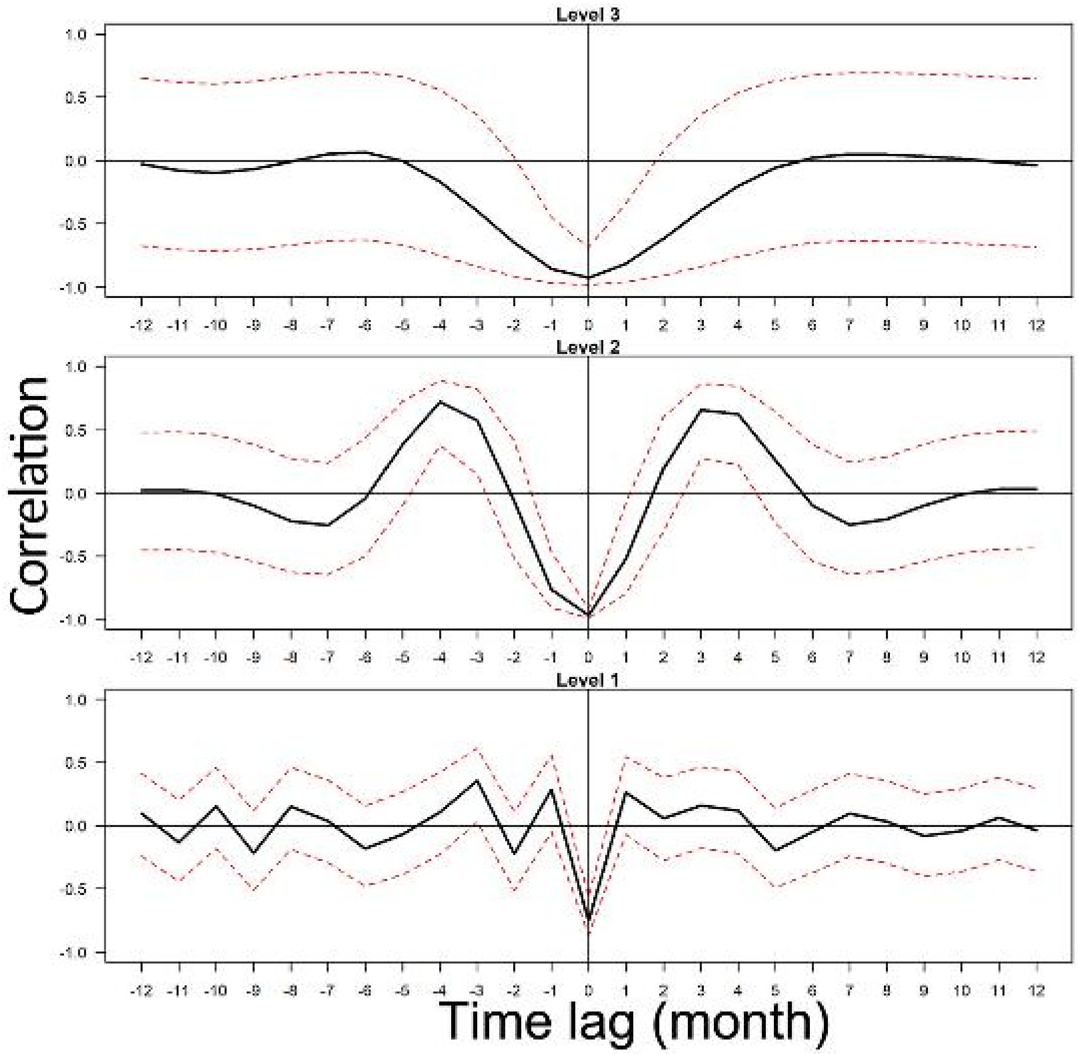

Appendix Figure 18. Wavelet cross-correlation between rubella cases and Google searches for “rubella,” 2012–2018, Kanagawa, Japan. Dotted line represents 95% confidence interval.

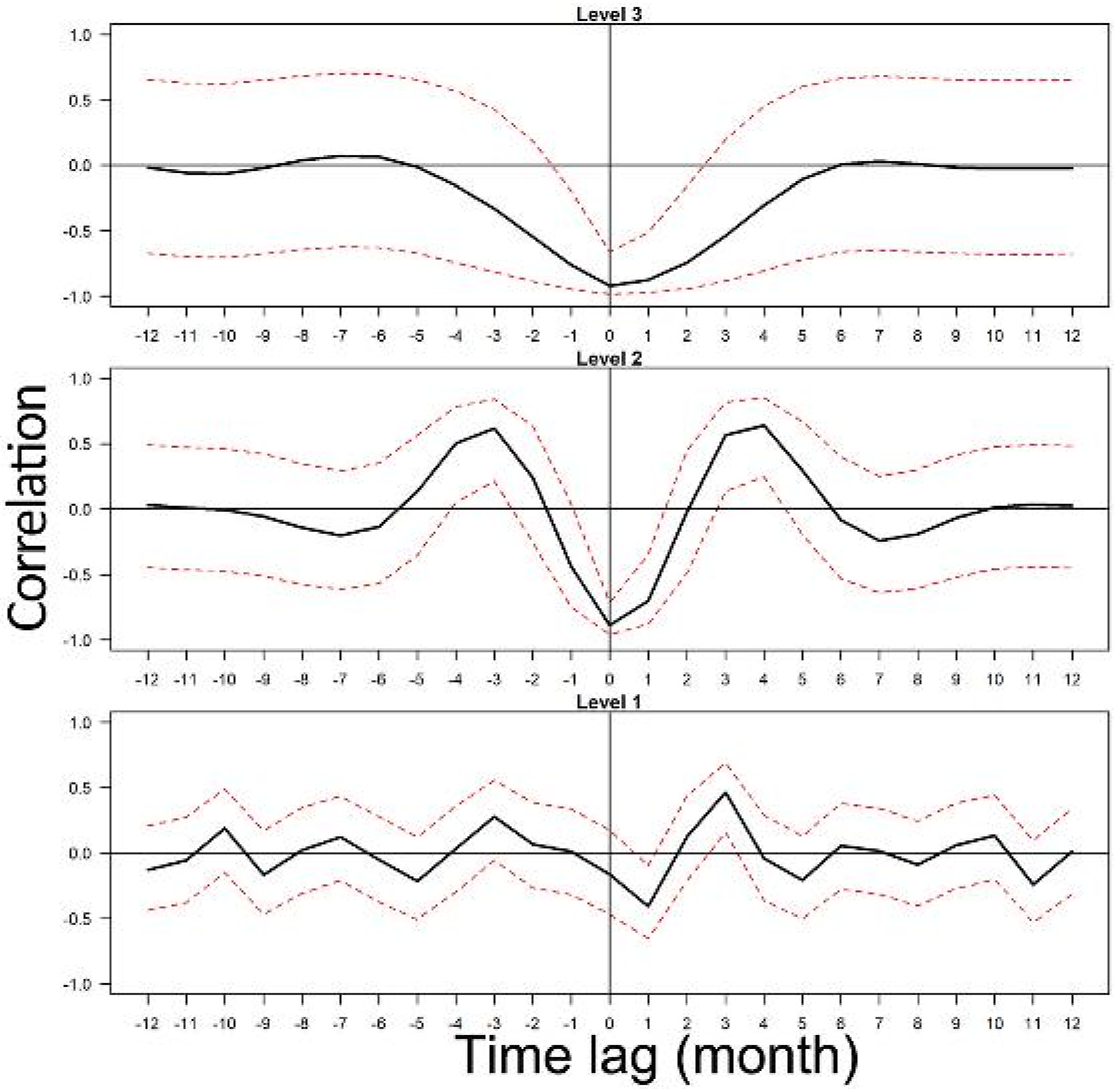

Appendix Figure 19. Wavelet cross-correlation between rubella cases and Google searches for "rubella," 2012–2018, Osaka, Japan. Dotted line represents 95% confidence interval.

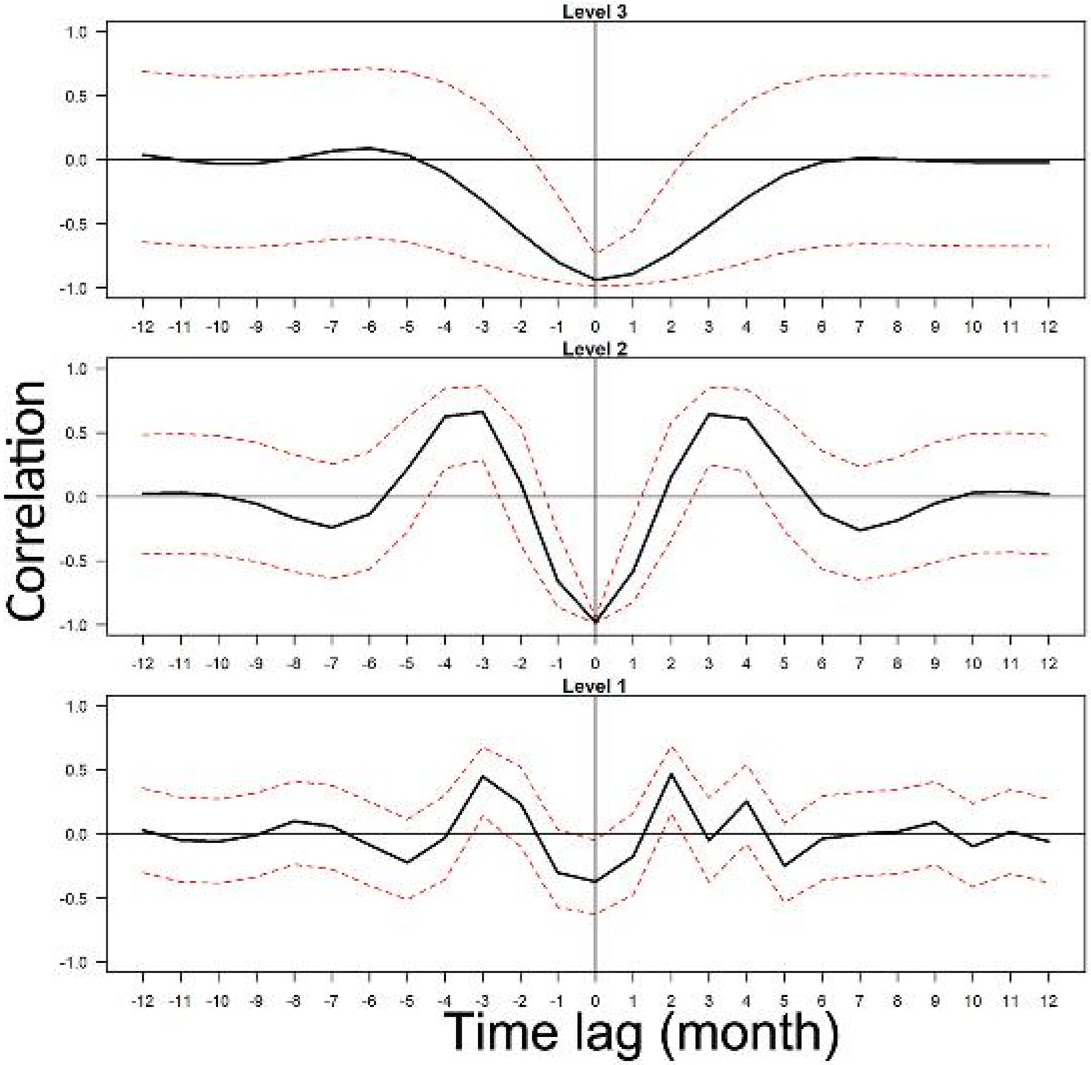

Appendix Figure 20. Wavelet cross-correlation between rubella cases and Google searches for "rubella," 2012–2018, Hyogo, Japan. Dotted line represents 95% confidence interval.
